# Structural and functional studies of the main replication protein NS1 of human parvovirus B19

**DOI:** 10.1093/nar/gkaf562

**Published:** 2025-06-26

**Authors:** Yixi Zhang, Boming Fan, Yanqing Gao, Jie Yang, Weizhen Zhang, Shichen Su, Linxi Li, Huili Li, Zhaorong Luo, Guangli Tang, Chenxi Wang, Xueting Zhang, Hehua Liu, Jianhua Gan

**Affiliations:** State Key Laboratory of Genetics and Development of Complex Phenotypes, Collaborative Innovation Center of Genetics and Development, Shanghai Sci-Tech Inno Center for Infection & Immunity, Department of Biochemistry and Biophysics, School of Life Sciences, Fudan University, Shanghai 200438, PR China; State Key Laboratory of Genetics and Development of Complex Phenotypes, Collaborative Innovation Center of Genetics and Development, Shanghai Sci-Tech Inno Center for Infection & Immunity, Department of Biochemistry and Biophysics, School of Life Sciences, Fudan University, Shanghai 200438, PR China; State Key Laboratory of Genetics and Development of Complex Phenotypes, Collaborative Innovation Center of Genetics and Development, Shanghai Sci-Tech Inno Center for Infection & Immunity, Department of Biochemistry and Biophysics, School of Life Sciences, Fudan University, Shanghai 200438, PR China; State Key Laboratory of Genetics and Development of Complex Phenotypes, Collaborative Innovation Center of Genetics and Development, Shanghai Sci-Tech Inno Center for Infection & Immunity, Department of Biochemistry and Biophysics, School of Life Sciences, Fudan University, Shanghai 200438, PR China; State Key Laboratory of Genetics and Development of Complex Phenotypes, Collaborative Innovation Center of Genetics and Development, Shanghai Sci-Tech Inno Center for Infection & Immunity, Department of Biochemistry and Biophysics, School of Life Sciences, Fudan University, Shanghai 200438, PR China; State Key Laboratory of Genetics and Development of Complex Phenotypes, Collaborative Innovation Center of Genetics and Development, Shanghai Sci-Tech Inno Center for Infection & Immunity, Department of Biochemistry and Biophysics, School of Life Sciences, Fudan University, Shanghai 200438, PR China; State Key Laboratory of Genetics and Development of Complex Phenotypes, Collaborative Innovation Center of Genetics and Development, Shanghai Sci-Tech Inno Center for Infection & Immunity, Department of Biochemistry and Biophysics, School of Life Sciences, Fudan University, Shanghai 200438, PR China; State Key Laboratory of Genetics and Development of Complex Phenotypes, Collaborative Innovation Center of Genetics and Development, Shanghai Sci-Tech Inno Center for Infection & Immunity, Department of Biochemistry and Biophysics, School of Life Sciences, Fudan University, Shanghai 200438, PR China; State Key Laboratory of Genetics and Development of Complex Phenotypes, Collaborative Innovation Center of Genetics and Development, Shanghai Sci-Tech Inno Center for Infection & Immunity, Department of Biochemistry and Biophysics, School of Life Sciences, Fudan University, Shanghai 200438, PR China; State Key Laboratory of Genetics and Development of Complex Phenotypes, Collaborative Innovation Center of Genetics and Development, Shanghai Sci-Tech Inno Center for Infection & Immunity, Department of Biochemistry and Biophysics, School of Life Sciences, Fudan University, Shanghai 200438, PR China; State Key Laboratory of Genetics and Development of Complex Phenotypes, Collaborative Innovation Center of Genetics and Development, Shanghai Sci-Tech Inno Center for Infection & Immunity, Department of Biochemistry and Biophysics, School of Life Sciences, Fudan University, Shanghai 200438, PR China; State Key Laboratory of Genetics and Development of Complex Phenotypes, Collaborative Innovation Center of Genetics and Development, Shanghai Sci-Tech Inno Center for Infection & Immunity, Department of Biochemistry and Biophysics, School of Life Sciences, Fudan University, Shanghai 200438, PR China; State Key Laboratory of Genetics and Development of Complex Phenotypes, Collaborative Innovation Center of Genetics and Development, Shanghai Sci-Tech Inno Center for Infection & Immunity, Department of Biochemistry and Biophysics, School of Life Sciences, Fudan University, Shanghai 200438, PR China; State Key Laboratory of Genetics and Development of Complex Phenotypes, Collaborative Innovation Center of Genetics and Development, Shanghai Sci-Tech Inno Center for Infection & Immunity, Department of Biochemistry and Biophysics, School of Life Sciences, Fudan University, Shanghai 200438, PR China

## Abstract

Parvovirus B19 (B19V) is a ubiquitous virus that can infect the majority of human population and cause erythema infectiosum, acute arthropathy, and many other diseases. The main replication protein NS1 plays a critical role in cell cycle arrest, transactivation of viral and host genes, and replication and package of B19V genome. Both DNA nicking and unwinding activities are required for the *in vivo* function of NS1, but the underlying basis is poorly understood. Here, we report extensive structural and biochemical studies of NS1, showing that NS1 can unwind various types of DNA substrates. The cryo-electron microscopy (cryo-EM) structures reveal the detailed mechanisms for ATP binding and hydrolysis, and DNA binding and unwinding by NS1. In addition to the SF3 HD domain, the C-terminal region is also required for double-stranded DNA (dsDNA) nicking by NS1. Unexpectedly, instead of enhancing, the dsDNA nicking activity of NS1 is negatively regulated by its DNA unwinding ability, suggesting that they likely function in different stages. This study advances our understanding of the structure and function of NS1 and other parvoviral replication proteins, such as the Rep proteins of adeno-associated virus.

## Graphical Abstract

**Graphical Abstract:**
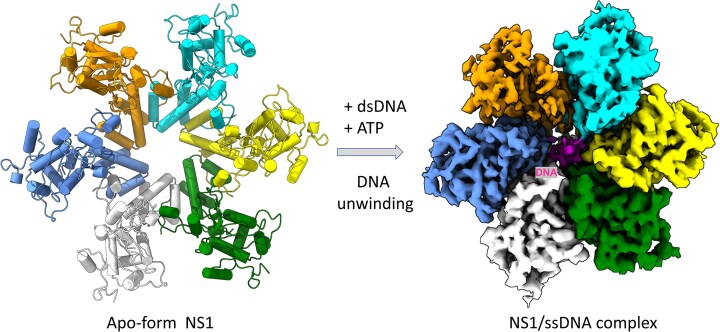

## Introduction

Parvovirus B19 (B19V) is a ubiquitous virus that can infect the majority of human population. Since its first discovery in 1975, B19V has been extensively studied [1, 2]. In addition to the common respiratory route, it was found that B19V can also be transmitted vertically from mother to fetus or through the transfusion of blood products and organ transplantation [3, 4]. B19V infection mainly occurs during childhood, but many adults are susceptible to B19V through respiratory droplet [5, 6]. Although most patients are asymptomatic, infection of B19V has been linked with a variety of illnesses in humans [7], such as hydrops fetalis [8], erythema infectiosum (also known as the fifth disease) in children [9], acute arthropathy in adults, and myocarditis in pediatric patients [10].

B19V is a small nonenveloped, single-stranded DNA (ssDNA) virus, which belongs to the *Erythrovirus* genus within the *Parvoviridae* family [11]. The genome of B19V is composed of 5596 nucleotides [12]. The central region encodes for six proteins, namely VP1, VP2, NS1, and three nonstructural proteins [13, 14]. VP2 is the major viral protein and can self-assemble into virus-like particles. In contrast to VP2, the viral protein VP1 only locates at the surface of the capsid. The nonstructural protein with a molecular weight of 11 kDa is involved in viral replication and interaction with host protein [15, 16], whereas the functions of another two nonstructural proteins are unclear.

The NS1 protein predominantly localizes to the nucleus of the infected cells. As the major replication protein, NS1 is required for the replication of the viral genome [16, 17] that follows a “rolling hairpin” mechanism (Supplementary Fig. S1) [18]. The hairpin structures are present at both ends of the viral genome. During replication, the 3′-end of one hairpin is first extended by a cellular polymerase, resulting in replication of the majority of the genome [19]. NS1 then cleaves at the terminal resolution site (TRS) of one strand, generating a new 3′-end that can be used for the subsequent synthesis of the viral DNA [20]. A previous study has identified a 67-nt replication origin (Ori) of B19V [21, 22]. In addition to TRS, the Ori also contains four NS1-binding elements, NSBE1–NSBE4, which are required for optimal B19V replication [23].

**Figure 1.**
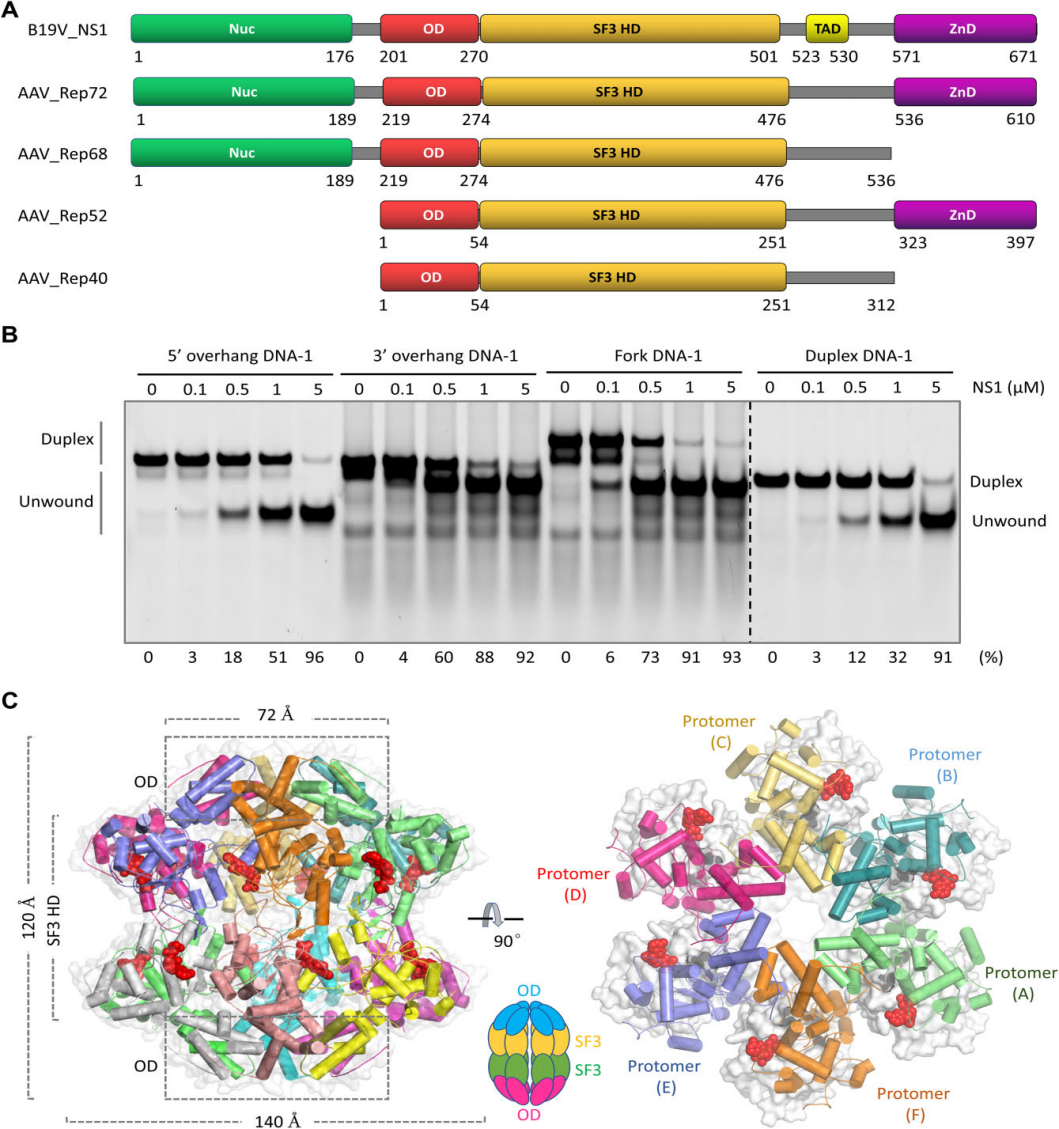
Domain architecture and DNA unwinding activity of B19V NS1 protein. (**A**) Domain architecture of B19V NS1 and the homologous proteins of AAV. (**B**) *In vitro* DNA unwinding assays catalyzed by NS1_2-570 protein. The detailed sequences of the four DNA substrates are available in Supplementary Fig. S3. The substrate unwinding percentage (%) is shown at the bottom of the gels. Experiments were repeated independently three times with similar results. (**C**) Overall folding and assembly of NS1 in the NS1_2-570/AMPPNP structure. A schematic view showing the assembly of NS1 dodecamer is shown at the bottom. In the left panel, the 12 NS1_2-570 protomers are shown as cartoon in different colors. In the right panel, the protomers in the upper hexamer are shown as cartoon, whereas the protomers in the lower hexamer are shown as surface in white. AMPPNP molecules are shown as red spheres.

NS1 is composed of 671 amino acids and is a multi-domain protein (Fig. 1A). Based on its similarity to other parvoviral replication proteins, such as Rep72 of the adeno-associated virus (AAV), NS1 is predicted to contain one nuclease domain (Nuc, aa 1–176), one oligomerization domain (OD, aa 201–270), one SF3 helicase domain (SF3 HD, aa 271–501), and one zinc-binding domain (ZnD, aa 571–671). Prior studies suggested that NS1 also contains one transactivation domain (TAD, aa 523–530), which is essential for the promoter transactivation activity of NS1 [24] and the arrest of the B19V infected cells at G2 phase [25]. With the help of Sp1 and Sp3 transcription factors, NS1 can regulate the expression of its own gene and several genes from the host [25–27]. It is known that the genome of B19V integrates into the host DNA. Similar to replication, the insertion of B19V genome also depends on the DNA nicking activity of NS1 [28].

Although NS1 plays multiple functions, the underlying basis is poorly understood. To date, only the apo-form Nuc domain (NS1_Nuc) structures are available [29, 30]. NS1_Nuc can bind B19V Ori in a sequence-dependent manner; however, it can only cleave ssDNA *in vitro* [21]. Since the target DNAs encountered by NS1 are double-stranded (ds), it was suggested that the other domains, especially the SF3 HD domain, are also critical for the *in vivo* functions of NS1. However, whether the SF3 HD domain is functional and how it cooperates with other domains are unclear. Here, we report structural and biochemical studies of NS1. Totally, four cryo-electron microscopy (cryo-EM) structures were determined, which revealed the detailed mechanisms for ATP binding and hydrolysis and DNA binding and unwinding by NS1. *In vitro* assays confirmed the functional importance of many SF3 HD domain residues in DNA unwinding. In addition to SF3 HD domain, the C-terminal region is also required for dsDNA nicking by NS1. Unexpectedly, instead of enhancing, the dsDNA nicking activity of NS1 is negatively regulated by its DNA unwinding ability. This study advances our understanding of the structure and function of NS1 and other parvoviral replication proteins.

## Materials and methods

### Plasmid construction

The codon-optimized complementary DNA of NS1 (Supplementary Table S1) was purchased from Shanghai Generay Biotech Co., Ltd, China. The target region was amplified by polymerase chain reaction (PCR), digested by BamHI and XhoI, and cloned into pET-His6-MBP-TEV-LIC or pET28a-Sumo cloning vectors. The recombinant plasmids of NS1 mutants were constructed by overlap PCR using the wild-type (WT) NS1 plasmid as template and primers listed in Supplementary Table S2. Sequences of WT and mutant plasmids were confirmed by DNA sequencing. The recombinant plasmids were then transformed into *Escherichia coli* Rosetta (DE3) competent cells for protein expression.

### Protein expression and purification

All NS1 proteins were expressed and purified using similar procedures. The cells were cultured at 37°C in LB medium supplemented with 50 mg/l kanamycin. When the OD_600_ reached 0.6–0.8, isopropyl-d-1-thiogalacto-pyranoside (final concentration of 0.2 mM) was added to induce protein expression. The cultures were incubated at 16°C for an additional 16–18 h. The cells were collected by centrifugation and resuspended in Buffer A (20 mM Tris–HCl, pH 8.0, 500 mM NaCl, 25 mM imidazole). The cells were lysed by high-pressure homogenization and centrifuged at 17 000 rpm for 1 h at 4°C. The supernatant was applied to 5-ml HisTrap™ HP column (Cytiva), and the target protein was eluted via AKTA purifier (GE Healthcare) using Buffer B (20 mM Tris–HCl, pH 8.0, 150 mM NaCl, 500 mM imidazole). The protein was then applied to 5-ml HiTrap™ Heparin HP column (Cytiva) and eluted by Buffer C (20 mM Tris–HCl, pH 8.0, 1 M NaCl, 25 mM imidazole). The target protein was pooled and treated with TEV protease prepared in the laboratory at 4°C for overnight. The sample was reloaded onto the HisTrap™ HP column. The target protein was collected and applied to Superose™ 6 Increase 10/300 GL column (Cytiva) equilibrated with gel filtration buffer (50 mM Tris–HCl, pH 8.0, 200 mM NaCl, 2 mM DTT). The purified protein was concentrated to 10 mg/ml and stored at −80 °C. The purity of the protein was analyzed by the SDS–PAGE (sodium dodecyl sulfate–polyacrylamide gelelectrophoresis) gels (Supplementary Fig. S2).

### Helicase assay

DNAs used in the helicase assays were prepared by annealing (Supplementary Fig. S3A). The mixtures were heated at 95°C for 3 min, followed by slow cooling to room temperature. Helicase assays with annealed substrates (0.1 μM) were performed in 50 mM Tris–HCl (pH 8.0), 150 mM NaCl, 2 mM DTT, 5% glycerol, 5 mM MgCl_2_, and 5 mM ATP or AMPPNP. To prevent the re-annealing of the unwound top and bottom strands, 20 μM nonlabeled bottom strand DNA was also included in the reaction system. Reaction mixtures were incubated at 37°C for 1 h and then digested with 2 mg/ml Proteinase K (Shanghai Sangon Biotech Co., Ltd) at room temperature for 1 h. Samples were loaded onto a 10% Tris-Borate-EDTA (TBE) polyacrylamide gel for electrophoresis at 4°C. The gel was imaged using Typhoon FLA 9000. Intensities of the substrate and product bands were quantified using ImageJ.

### DNA cleavage assays

To prepare the DNAs (Supplementary Fig. S3A) used in the cleavage assays, the 5′-FAM-labeled strand was mixed with the unlabeled strand and annealed. The annealed DNA was mixed with B19V NS1 protein at final concentration of 0.1 and 5 μM, respectively. The mixture was incubated at 37°C in 50 mM Tris–HCl (pH 8.0) and 150 mM NaCl. The reactions were quenched with the 2 mg/ml Proteinase K (Shanghai Sangon Biotech Co., Ltd) at various time points. Samples were loaded onto prewarmed 16% denaturing PAGE gels. The gel was visualized using Typhoon FLA 9000. Intensities of the substrate and product bands were quantified using ImageJ.

### Electrophoretic mobility shift assay experiment

Different quantities of WT or mutant proteins of NS1_2-570 ranging from 0 to 4.8 μM were mixed with 0.1 μM duplex DNA-1 in buffer (50 mM Tris–HCl, pH 8.0, 150 mM NaCl, 2 mM DTT, and 5% glycerol). The 5′-end of the top strand of the DNA was FAM-labeled. The total volume of the reaction mixture was 10 μl. The samples were incubated on ice for 1 h and then analyzed on 1.2% agarose gels in 0.5× TBE buffer (45 mM Tris, 45 mM boric acid, 1 mM ethylenediaminetetraacetic acid, pH 8.0). The gel was visualized using Typhoon FLA 9000. Intensities of the substrate and product bands were quantified by ImageJ. The percentage of binding, for each protein concentration, was calculated. Data were then fitted to the equation *Y*= *B*_max_*X^*h*/(*K*_d_^*h* + *X*^*h*) using nonlinear regression (curve fit) in GraphPad Prism. The dissociation constants (*K*_d_) were determined from the regression curve.

### Cryo-EM sample preparation and data collection

All proteins used for cryo-EM analysis were dissolved in gel filtration buffer. For the dsDNA-bound structure, the two strands of the fork DNA-2 (Supplementary Fig. S3B) were mixed, heated at 95°C for 3 min, and slowly cooled down to room temperature. Complexes were assembled with 60 μM protein and 10 μM fork DNA-2 and incubated at 4°C for 1 h. The mixture was then injected into the Superose™ 6 Increase 3.2/300 GL column, and the peak was collected. Both MgCl_2_ and AMPPNP were added to the mixture to a final concentration of 5 mM. To capture the reaction at different states, the two strands of the overhang DNA-2 (Supplementary Fig. S3B) were mixed and annealed. The mixture composed of 18 μM protein, 9 μM overhang DNA-2, 5 mM MgCl_2_, and 5 mM of ATP was incubated at 37°C for 15 min. AMPPNP was added to terminate the reaction at a final concentration of 10 mM.

For the dsDNA-bound NS1_2-570 structure, an aliquot of 2.5 μl of sample was applied to a copper R 1.2/1.3 300 mesh grid (Beijing Zhongjingkeyi Technology), which was freshly glow-discharged for 30 s. The grids were blotted with a couple of 55-mm filter papers (Ted Pella) at 22°C and 100% humidity for 5 s, flash-frozen in liquid ethane using the FEI Vitrobot Mark IV. For the DNA-free and ssDNA-bound NS1_2-570 structures, an aliquot of 3 μl of sample was applied to a gold R 1.2/1.3 300 mesh grid (Quantifoil), which was freshly glow-discharged for 60 s. The sample for the NS1_200-501/AMPPNP structure was applied to a copper R 1.2/1.3 300 mesh grid (Quantifoil). Other conditions were the same as the ssDNA-bound NS1_2-570 complex.

The data for all the NS1_2-570 structures were collected using EPU software (Thermo Fisher) on a Krios G4i electron microscope (Thermo Fisher) operating at 300 kV and equipped with Falcon 4i with SelectrisX direct electron detector. Images were recorded at a nominal magnification of 130 000, corresponding to a pixel size of 0.959 Å per pixel and defocus values ranged from −1.2 to −2.0 μm. Data for the NS1_200-501 structure were collected using EPU software (Thermo Fisher) on a Titan Krios electron microscope (Thermo Fisher) operating at 300 kV and equipped with Gatan K3 direct electron detector. Images were recorded at a nominal magnification of 64 000, corresponding to a pixel size of 1.1 Å per pixel and defocus values ranged from −1.3 to −1.8 μm.

### Electron microscopy data processing, model building, and validation

For all structures, the cryo-EM images were processed using similar steps, dose-weighted, and summed with MotionCor2 [31]. The contrast-transfer function parameters for each micrograph were determined using CTFFIND4 [32]. Single particles were picked and processed using RELION [33]. For all the NS1_2-570 structures, after several rounds of 2D classification, the *ab initio* reconstruction was performed in CryoSPARC [34]. Particles with ordered density and clear structural features were selected for further homogeneous refinement and 3D classification in CryoSPARC. Local resolution distribution was evaluated using CryoSPARC. For the NS1_200-501 structure, all processes were completed in RELION-3.0.

The model of the NS1 monomer was predicted by AlphaFold2 [35] and docked into the EM 3D density maps using the program ChimeraX [36]. Model adjustment was done manually in ChimeraX and COOT [37]. The resulting models were refined against the EM map by PHENIX [38] in real space with secondary structure and geometry restraints. The final models were validated using the PHENIX software package. The model statistics are summarized in Supplementary Table S3.

## Results

### NS1_2-570 can efficiently unwind various types of DNAs

To unravel the basis underlying the function of NS1, we designed various NS1 constructs. Due to insolubility or difficulty in expression, no full-length protein could be obtained, but the N-terminal His-MBP-tagged NS1_2-570 protein can be expressed and purified to homogeneous (Supplementary Fig. S2A). The SF3 HD domain of NS1 belongs to the SF3 helicase superfamily, which unwinds DNA with 3′→5′ polarity [39]. To clarify whether NS1 possesses DNA unwinding activity, we synthesized four different DNAs. As depicted in Supplementary Fig. S3A, the 5′ overhang DNA-1, 3′ overhang DNA-1, fork DNA-1, and duplex DNA-1 all contain 48 base pairs, which are identical to the reported B19V Ori. Except for the duplex DNA-1, all other DNAs carry overhangs at their 5′- and/or 3′-ends.

Using the purified NS1_2-570 protein and the synthesized DNAs, we performed *in vitro* DNA unwinding assays (Fig. 1B). Compared to the 5′ overhang DNA-1 and duplex DNA-1, the 3′ overhang DNA-1 and fork DNA-1 were more efficiently unwound by NS1_2-570 at a concentration of 0.5 or 1.0 μM. In the presence of 5 μM NS1_2-570, >90% unwinding occurred to all the DNAs. These observations suggested that NS1 can unwind DNA and does not have a strong preference for the substrates, which is different from many known helicases including Mpox E5 protein that only unwinds forked DNAs [40].

### Cryo-EM structure of NS1_2-570 in complex with AMPPNP

Upon the confirmation of the DNA unwinding activity, we then analyzed the oligomerization state of NS1_2-570 by gel-filtration chromatography (Supplementary Fig. S2B). Two major peaks are observed. The first one is eluted at the void volume of the column and the second one is eluted at a volume of 12.1 ml, corresponding to a molecular weight of ∼830 kDa. The theoretical molecular weight of the NS1_2-570 monomer is 62 kDa. Unlike the E1 helicase from papillomavirus [41], Mpox E5 protein [40], and many other SF3 family members that assemble as hexamer, the gel filtration profile suggests that NS1_2-570 exists as higher oligomer.

To reveal how NS1_2-570 assembles, we performed cryo-EM study (Supplementary Fig. S4) and solved one NS1_2-570/AMPPNP binary complex. As depicted in Fig. 1C and Supplementary Fig. S5A, NS1_2-570 exists as a dodecamer in the structure. The dodecamer has a height of 120 Å and it is formed by two hexamers. The overall conformations of the two hexamers are virtually identical (Supplementary Fig. S5B), supported by the very low root mean square deviation (RMSD) value (0.5 Å). Each hexamer adopts a double-layered ring-shaped conformation; the top and bottom layers are formed by the OD and SF3 HD domains, respectively. Compared to that of the top layer, the diameter of the bottom layer (140 Å) is much wider.

The NS1_2-570/AMPPNP complex was determined at an overall resolution of 2.75 Å (Supplementary Table S3). Since no density was observed for the residues within the 2–201, 279–285, and 500–570 regions, they were not modeled in the final structure. In contrast, clear density was produced for all other residues from the OD and SF3 HD domains (Supplementary Fig. S6). The OD domain is mainly composed of four α-helices (α1–α4), whereas the SF3 HD domain is of α/β fold in nature (Supplementary Fig. S7). There are seven β-strands within the SF3 HD domain. The strands β1–β4 and β7 form one flat parallel β-sheet in the center, flanked by β5–β6 and α10–α12 on one side and by α5–α9 and α13–α15 on the opposite side. The hexamerization of NS1 is mainly mediated by the OD domain. The 202–209 region of NS1 forms a loop and functions as a latch by interaction with the neighboring protomer (Supplementary Fig. S8A and B). The main-chain O atom of Val203 forms one hydrogen bond (H-bond) interaction with the side-chain NE1 atom of Trp222. The main N atom of Phe205 forms H-bond interaction with the OD2 atom of Asp238; the side chain of Phe205 forms hydrophobic interactions with the side chains of Met219 and Trp222. Hydrophobic interactions are also formed by Phe264 and Leu269 of one protomer and Lys235, Leu236, and Phe239 of the neighboring protomer (Supplementary Fig. S8C). The NS1 hexamer is further stabilized by the hydrophobic interaction mediated by Phe253 and His249, and the H-bond interactions mediated by Ser246, Ser247, Gln254, and Ser257 (Supplementary Fig. S8D).

The NS1 protomers are related by six-fold axis in the hexamer (Supplementary Fig. S9A). Unlike the OD domain, the SF3 HD domains are loosely arranged. Only two H-bond interactions are observed, which are mediated by Arg394 and Lys398 of one protomer and Asn352 and Asp362 of the neighboring protomer. The 393–400 region forms a short helix; its backbone packs against the side chain of Trp353 (Supplementary Fig. S9B). The dodecamerization of NS1_2-570 is mediated by the linker connecting the β5 and β6 strands (Supplementary Fig. S9C and D). The linker residue Asn425 forms two H-bond interactions, one with Glu356 and the other with Ile375. One additional H-bond interaction is formed between Thr427 and Glu373.

### ATP binding and hydrolysis are critical for DNA unwinding by NS1

**Figure 2.**
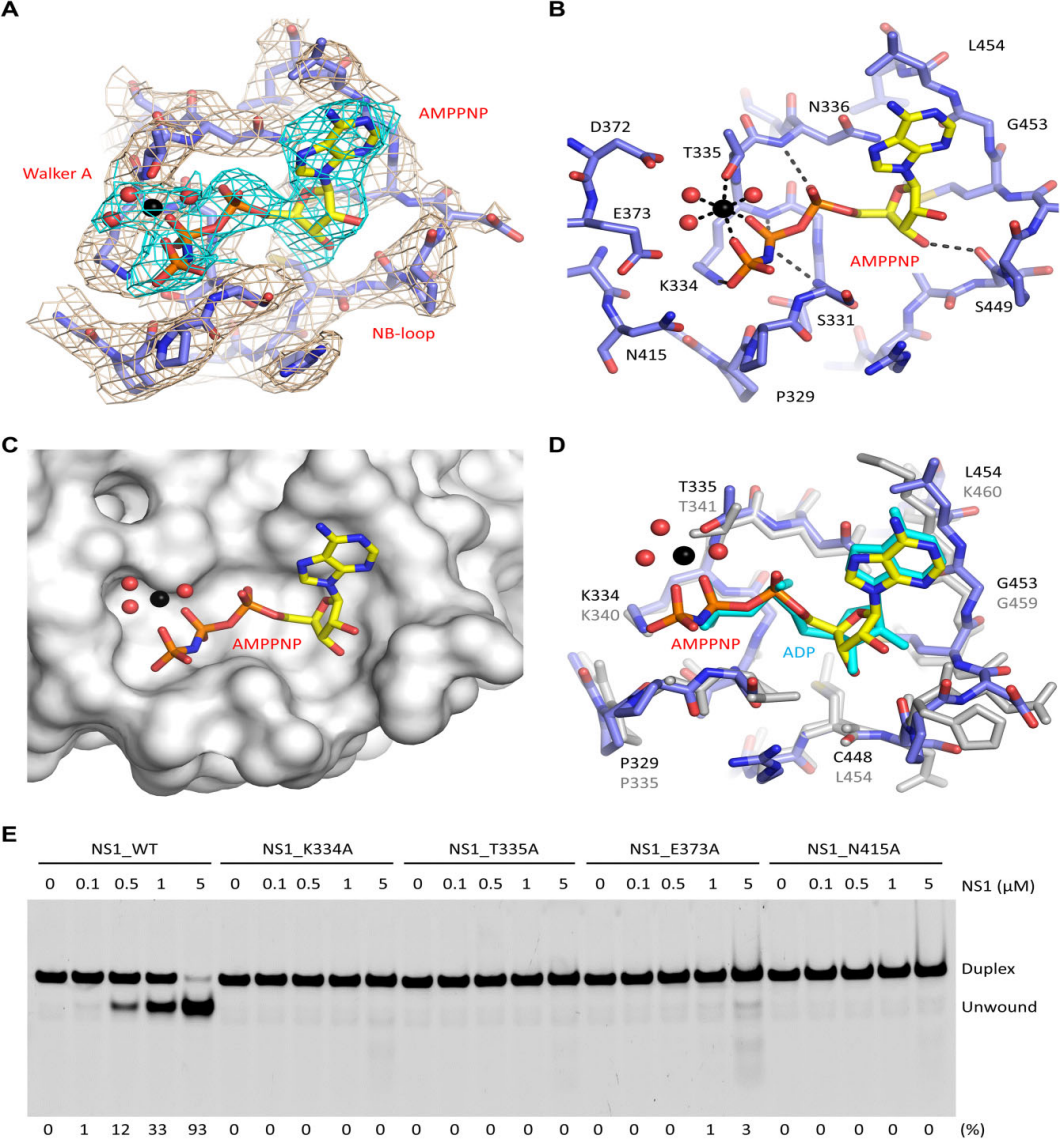
AMPPNP recognition in the NS1_2-570/AMPPNP structure. (**A**) Density maps of AMPPNP and the surrounding residues. (**B**) The detailed interactions between AMPPNP and NS1_2-570. (**C**) Stick-and-surface presentation showing the open conformation of the AMPPNP-binding pocket. (**D**) Superposition of AMPPNP and ADP bound in the NS1_2-570/AMPPNP structure and the AAV2 Rep40/ADP complex (PDB_ID: 1U0J), respectively. (**E**) *In vitro* assays showing the effects of mutation of AMPPNP-interacting residues on duplex DNA-1 unwinding by NS1. The substrate unwinding percentage (%) is shown at the bottom of the gels. Experiments were repeated independently three times with similar results. For the Rep40/ADP complex, ADP and Rep40 are colored in cyan and gray, respectively. For the NS1_2-570/AMPPNP structure, AMPPNP is shown as sticks in atomic color (C, yellow; N, blue; O, red; P, orange). Mg^2+^ and water molecules are shown as spheres in black and red, respectively.

ATP is a known factor of many helicases. To reveal how ATP is recognized by NS1, the non-hydrolyzable analogue AMPPNP was utilized in the sample preparation. In the NS1_2-570/AMPPNP complex, one AMPPNP molecule is captured at the characteristic Walker A region of each NS1 protomer (Fig. 2A). The conformation of the AMPPNP is stabilized by various types of interactions. As depicted in Fig. 2B, the α- and β-phosphate groups of AMPPNP form H-bond interactions with the main chains of Ser331, Lys334, and Asn336. The γ-phosphate H-bonds with the side chain of Lys334. Both β- and γ-phosphate groups coordinate with an Mg^2+^ cation, which is six-coordinated. In addition to the phosphate groups, the Mg^2+^ also coordinates with the side chain of Thr335 and three water molecules. The 3′-OH group of the sugar pucker forms one H-bond interaction with the main-chain O atom of Ser449. The nucleobase of AMPPNP points toward the 449–454 region, forming stacking interactions with the side chains of Asn336 and Leu454.

As observed in Mpox E5 [40] and many other helicase structures [42], the ATP-binding pocket usually adopts a closed conformation. In contrast, the ATP-binding pocket is widely open in the NS1_2-570/AMPPNP complex (Fig. 2C). One crystal structure of AAV2 Rep40 protein (PDB_ID: 1U0J) was previously reported [43], which captured an ADP molecule, the hydrolysis product of ATP, in the Walker A region. The ADP-binding pocket also showed an open conformation in the structure (Fig. 2D), which could not explain the ATP preference of the Rep proteins [44]. Except the Walker A motif, previous studies revealed that some other structural features are also conserved in the SF3 helicase superfamily, such as the Walker B motif and the sensor motif [41].

Guided by our structural observations (Fig. 2B) and previous reports, we constructed and purified four NS1_2-570 single-point mutants, including K334A, T335A, E373A, and N415A (Supplementary Fig. S2C). Although Glu373 and Asn415 are not involved in direct AMPPNP binding in the NS1_2-570 structure, their corresponding residues could improve the activities of other SF3 helicases by sensing or facilitate the hydrolysis of ATP [39, 41]. Using duplex DNA-1 (Supplementary Fig. S3A) and the purified proteins, we performed *in vitro* DNA unwinding assays. As depicted in Fig. 2E, the DNA unwinding activity of the E373A mutant is much weaker than that of WT protein. At a concentration of 5 μM, the E373A mutant can only unwind ∼3% of the DNA substrate. No DNA unwinding activity could be observed for either the K334A, the T335A, or the N415A mutant. To better understand DNA unwinding by NS1, a time course analysis was performed (Supplementary Fig. S10). In the presence of 5 μM NS1_2-570, over 50% duplex DNA-1 was unwound within 1 min. About 90% duplex DNA-1 was unwound at 5 min. In contrast, no obvious DNA unwinding activity was observed for the K334A mutant during the time course analysis. Taken together, these observations suggested that NS1 may follow a conserved mechanism in ATP hydrolysis, which is critical for its DNA unwinding activity [39].

### ssDNA is recognized by the SF3 HD domain of NS1

**Figure 3.**
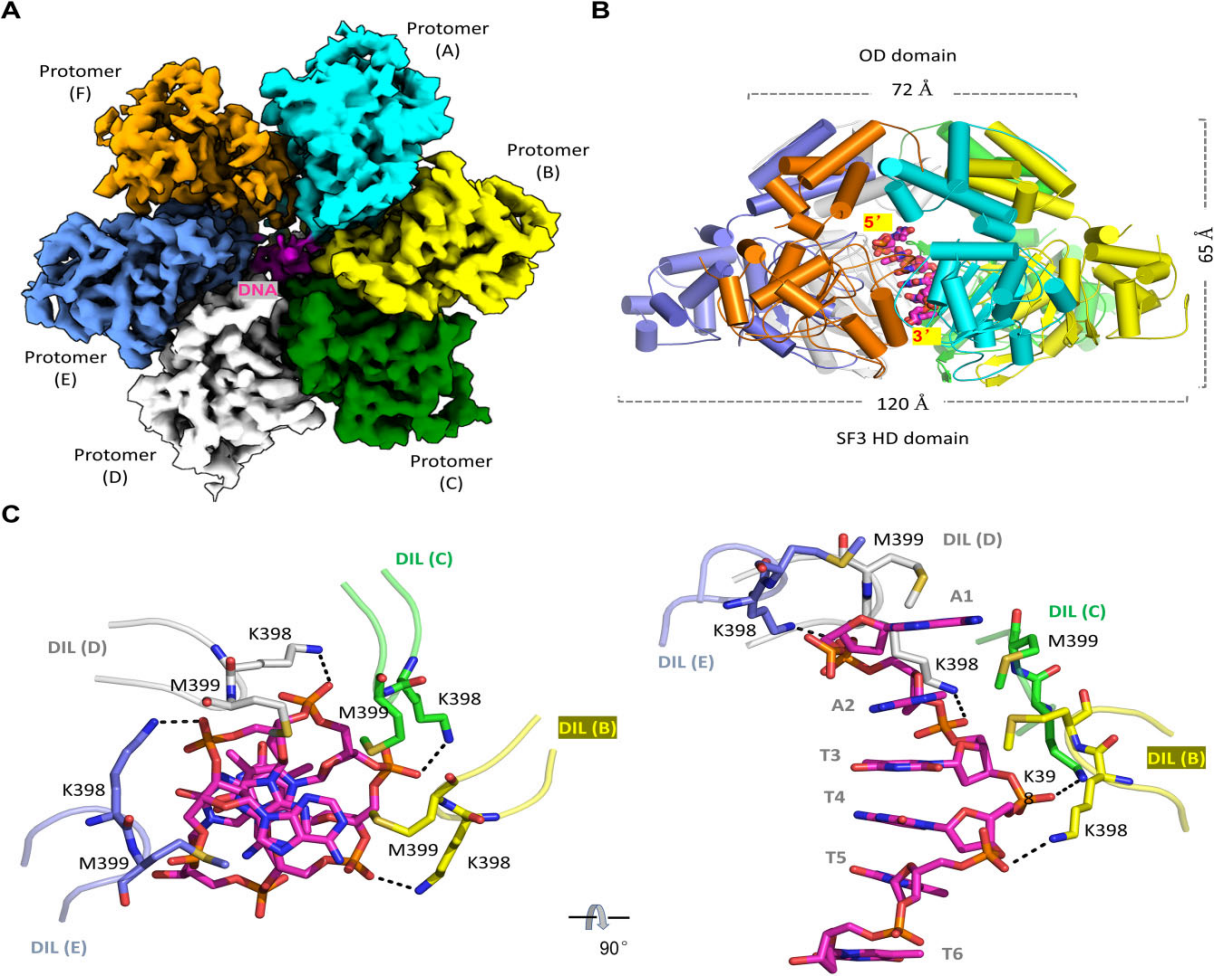
ssDNA binding by NS1_2-570. (**A**) The final density maps of the NS1_2-570/ssDNA/AMPPNP structure. (**B**) The overall conformation and assembly of the NS1_2-570/ssDNA/AMPPNP structure. (**C**) The detailed interactions between ssDNA and NS1_2-570. The C-atoms of the ssDNA are colored magenta. The C-atoms of the NS1_2-570 protomers A–F are colored in cyan, yellow, green, gray, light blue, and orange, respectively.

To reveal how NS1 unwinds DNA, a DNA with overhangs at both ends, the overhang DNA-2 (Supplementary Fig. S3B), was designed and included in the cryo-EM sample. One ternary complex was determined at an overall resolution of 3.3 Å (Supplementary Fig. S4 and Supplementary Table S3). Since the dsDNA region is disordered and only the ssDNA portion and AMPPNP are observed (Supplementary Fig. S11), the structure was termed as NS1_2-570/ssDNA/AMPPNP complex hereafter. Instead of dodecamer, NS1_2-570 exists as hexamer in the NS1_2-570/ssDNA/AMPPNP complex (Fig. 3A and B). The height of the NS1 hexamer is 65 Å; the diameters of the top layer and bottom layer of the ring are 72 and 120 Å, respectively.

The ssDNA mainly interacts with the SF3 HD domain with the 5′-end pointing toward the OD domain (Fig. 3B). The orientation of the DNA is identical to these captured by many hexameric helicases, such as the E1 helicase [41] and the eukaryotic CMG helicase [45, 46]. In addition to 3′→5′ unwinding polarity, this observation also suggested that NS1 processes the DNA in an N-first orientation. In the NS1_2-570/ssDNA/AMPPNP structure, the ssDNA adopts B-form-like conformation; its backbone phosphate groups form several H-bond interactions with the side chains of Lys398 of NS1 protomers B–E (Fig. 3C). The side chains of Met399 of protomers B–E point toward the sugar puckers of ssDNA, forming hydrophobic stacking interactions. In contrast, no interaction forms between ssDNA and the SF3 HD domains of protomers A and F.

### ssDNA binding leads to large conformational changes of NS1

**Figure 4.**
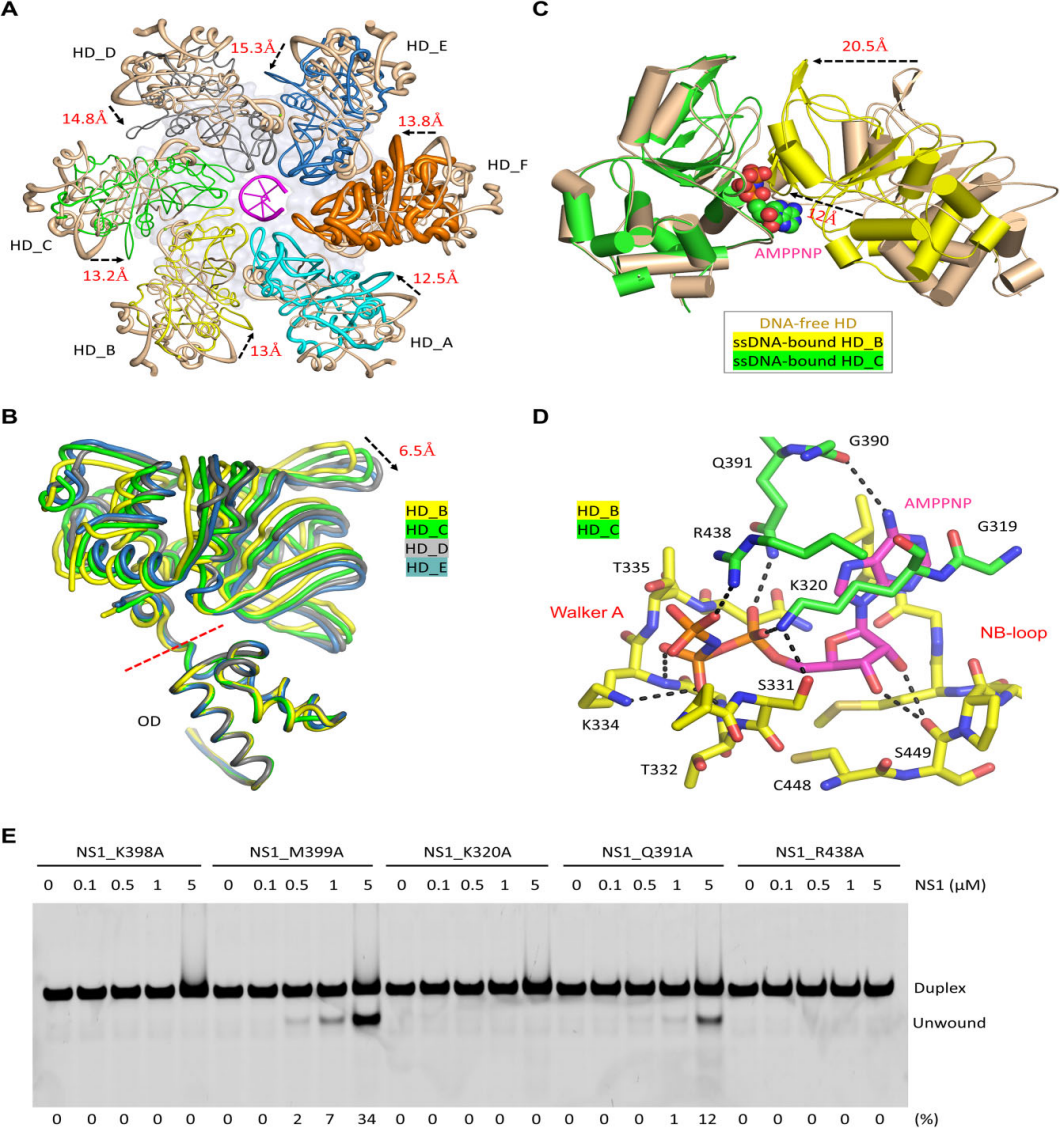
Conformational changes induced by ssDNA binding. (**A**) Superposition of DNA-free and ssDNA-bound NS1_2-570 structures, which are presented in sausage views based on the *B* factors. (**B**) Superposition of NS1_2-570 protomers B–E in the ssDNA-bound structure. (**C**) Superposition showing the shifting of the neighboring SF3 HD domain. (**D**) The detailed interactions between AMPPNP and NS1_2-570 in the ssDNA-bound structure. (**E**) *In vitro* assays showing the effects of the ssDNA- or AMPPNP-interacting residue mutations on duplex DNA-1 unwinding by NS1. The substrate unwinding percentage (%) is shown at the bottom of the gels. Experiments were repeated independently three times with similar results. The NS1_2-570 protomers are colored in pink in the DNA-free structure. For the ssDNA-bound structure, the C-atoms of protomers A–F are colored in cyan, yellow, green, gray, light blue, and orange, respectively. The C-atoms of AMPPNP are colored in magenta.

The oligomerization state of NS1 is very different in the DNA-free binary complex and the ssDNA-bound ternary complex; we then wondered whether the detailed conformation of NS1 is also different in the two structures. To this end, we performed careful structural comparison. As depicted in Supplementary Fig. S12A, the overall conformation and assembly of the OD domains are very similar in the two structures, supported by the very low RMSD value (0.3 Å). However, unlike the DNA-free structure, the SF3 HD domains adopt asymmetric assembly and tilt toward the central channel in the ssDNA-bound structure (Fig. 4A). Compared to the protomer A, more significant shifting occurs to other SF3 HD domains, especially those of protomers D and E. When aligned based on the OD domain, the SF3 HD domain undergoes continuous shifting in protomers B–E (Fig. 4B). When compared to the NS1 protomer in the DNA-free structure, there is >25° rotation between the OD and SF3 HD domains in the ssDNA-bound structure (Supplementary Fig. S12B).

Direct superposition showed that ssDNA binding also leads to large conformational change at the 393–407 region (Supplementary Fig. S12C), which folds into a short α-helix in the DNA-free structure (Supplementary Fig. S9B) and a β-sheet in the DNA-bound structures, respectively. Since Lys398 and Met399 involve in ssDNA binding (Fig. 3C), the 393–407 region is termed DNA-interacting loop (DIL). Conformational change also occurs to Loop-1 (aa 349–365), which stabilizes the conformation of DIL in the ssDNA-binding structure.

Due to their rotation and tilting, the gaps between the SF3 HD domains are significantly narrowed in the ssDNA-bound structure (Fig. 4C). The protomer B residues Gly319, Lys320, Gly390, Gln391, and Arg438 reside close to the AMPPNP bound by the protomer C (Fig. 4D). The side chain of Arg438 forms one H-bond interaction with the γ-phosphate of AMPPNP, and the β-phosphate interacts with the side chains of Lys320 and Gln391. The backbones of Gly319 and Lys320 hydrophobically stack with the nucleobase of AMPPNP. The N6 atom of the AMPPNP forms one H-bond interaction with the main-chain O atom of Gly390, which may contribute to the ATP preference of NS1. Together with the Walker A motif and the 449–454 region of protomer C, these residues form a close binding pocket for AMPPNP. The close binding pockets are also formed between protomers C and D, D and E, and E and F in the DNA-bound structure.

Directed by the structural observations (Figs 3C and 4D), we constructed and purified five NS1_2-570 single-point mutants, including K320A, Q391A, R438A, K398A, and M399A (Supplementary Fig. S2C). Using duplex DNA-1 as substrates, we performed *in vitro* DNA unwinding assays. As depicted in Fig. 4E, no obvious DNA unwinding activity could be observed for the K320A, the K398A, or the R438A mutant. The Q391A and the M399A mutants can unwind DNA, but are less efficient than the WT protein (Fig. 1B). All together, these observations suggested that the five DNA or ATP binding residues are important for the DNA unwinding function of NS1.

### dsDNA is bound by the OD domain of NS1

**Figure 5.**
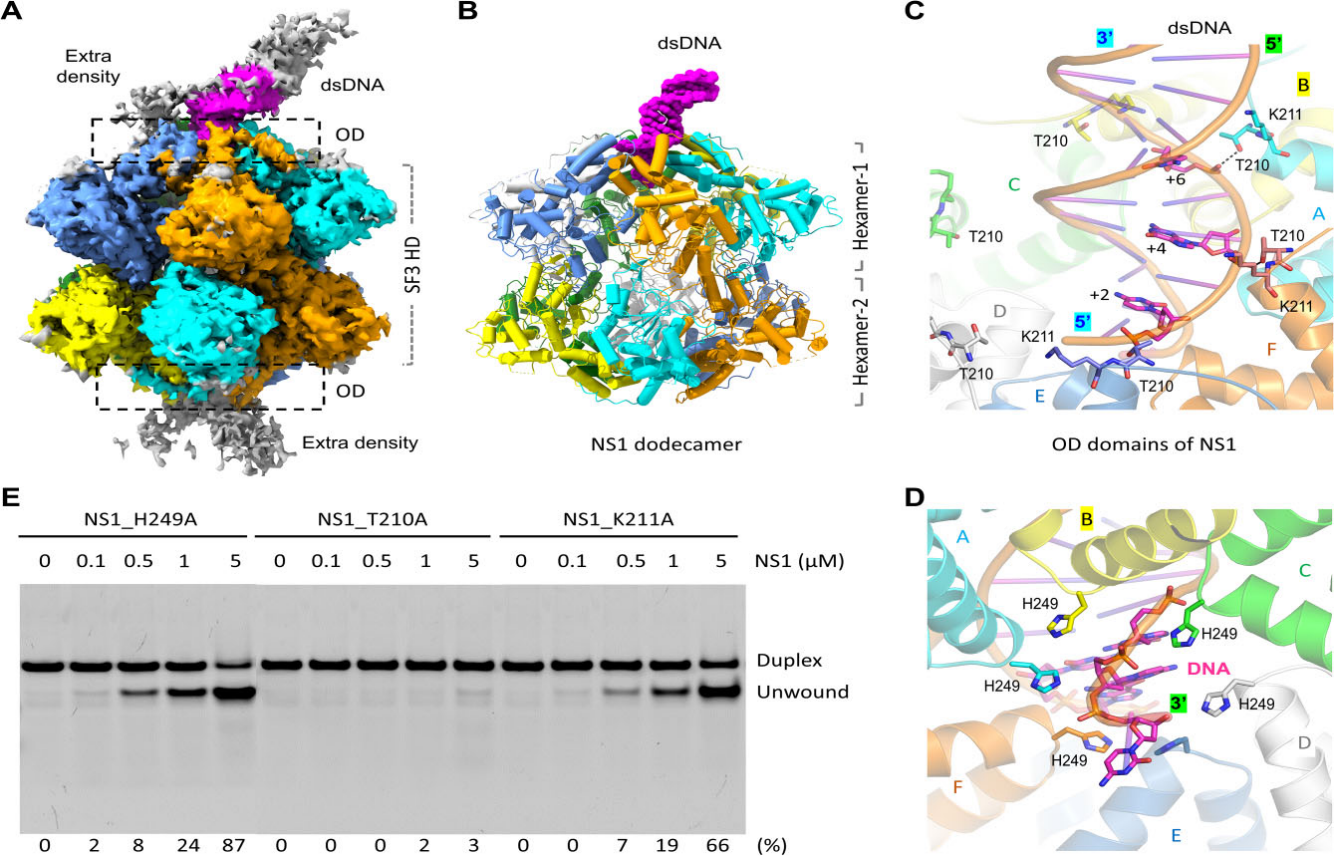
dsDNA binding by NS1_2-570. (**A**) The final density maps of the NS1_2-570/dsDNA/AMPPNP structure. (**B**) The overall conformation and assembly of the NS1_2-570/dsDNA/AMPPNP structure. (**C**, **D**) The detailed interactions between dsDNA and NS1_2-570 observed in the NS1_2-570/dsDNA/AMPPNP structure. (**E**) *In vitro* assays showing the effects of mutation of dsDNA-interacting residues on duplex DNA-1 unwinding by NS1. The substrate unwinding percentage (%) is shown at the bottom of the gels. Experiments were repeated independently three times with similar results. The C-atoms of dsDNA are colored in magenta. The C-atoms of protomers A–F are colored in cyan, yellow, green, gray, light blue, and orange, respectively.

As indicated by the above structural and biochemical analysis, NS1 can unwind dsDNA. However, no dsDNA portion was observed in the NS1_2-570/ssDNA/AMPPNP structure. To reveal how NS1 recognizes DNA duplex, we performed further cryo-EM study using fork DNA-2 (Supplementary Fig. S3B) and solved one NS1_2-570/dsDNA/AMPPNP ternary complex at an overall resolution of 3.43 Å (Supplementary Fig. S13 and Supplementary Table S3). Like the NS1_2-570/AMPPNP structure, NS1 forms dodecamer in the dsDNA-bound structure (Fig. 5A and B). As supported by the low RMSD value (0.8 Å), dsDNA binding does not cause significant change to the conformation and overall assembly of NS1 in the dsDNA-bound structure (Supplementary Fig. S14).

The density maps suggested that dsDNA could be bound by both NS1 hexamers, hexamer-1 and hexamer-2 (Fig. 5A). However, due to the density associated with hexamer-2 is relatively weak, only the DNA bound by hexamer-1 is modeled in the final structure (Fig. 5B and Supplementary Fig. S15). The DNA adopts regular B-form-like conformation; it is inclined toward one side of the ring formed by the OD domains. As depicted in Fig. 5C, the side chains of Thr210 of NS1 protomers A, E, and F point toward the DNA, forming H-bond interactions with the backbone phosphate groups of +6, +2, and +4 nucleotides, respectively. Instead of interacting with the phosphate group, the side chain of Lys211 points toward the major groove of the DNA. The His249 residue of the OD domain locates at the DNA entry site. In the dsDNA-bound NS1 structure, the side chain of the His249 wraps around the DNA backbone from the major or minor groove sides (Fig. 5D). The 3′-end nucleotide of the DNA is inserted into the central ring formed by the OD domains, further confirmed the 3′→5′ unwinding polarity of NS1.

Directed by the structural observations, we constructed and purified three NS1_2-570 mutants, including T210A, K211A, and H249A (Supplementary Fig. S2C), and performed *in vitro* DNA unwinding assays using duplex DNA-1 as substrates. As depicted in Fig. 5E, the K211A and H249A mutants can efficiently unwind the DNA, but the DNA unwinding activity of the T210A mutant is significantly weakened. To further investigate the functional importance of the dsDNA-interacting residues, *in vitro* electrophoretic mobility shift assays were performed. As depicted in Supplementary Fig. S16, the DNA binding affinities of the K211A and H249A mutants are comparable to that of WT NS1_2-570 protein, but the DNA binding affinity of the T210A is significantly lowered. Taken together, these observations suggested that Thr210 plays more important role in DNA binding, which is essential for the DNA unwinding activity of NS1.

### The 200–501 region of NS1 is sufficient for DNA unwinding

Although NS1_2-570 was used in above cryo-EM studies, only the OD and SF3 HD domains are observed in the structures (Figs 1C, 3A and B, and 5A and B). Based on these observations, we constructed and purified the NS1_200-501 protein, which is composed of the OD and SF3 HD domains (Supplementary Fig. S17A and B). The NS1_200-501 protein is eluted at a volume of 10.6 ml on the Superdex™ 200 Increase 10/30 GL column. Like NS1_2-570, the size-exclusion chromatography profile suggests that NS1_200-501 exists as dodecamer. To further verify the assembly of NS1_200-501, we also performed cryo-EM study and solved one NS1_200-501/AMPPNP complex at an overall resolution of 3.5 Å (Supplementary Fig. S18 and Supplementary Table S3). In the complex structure, NS1_200-501 assembles as dodecamer. Indicated by the low RMSD value (0.7 Å), the conformation and assembly of the NS1_200-501/AMPPNP complex are very similar to that of the NS1_2-570/AMPPNP structure (Supplementary Fig. S17C).

In addition to WT protein, we also constructed two NS1_200-501 mutant proteins, K334A and 3G (Supplementary Fig. S17B). In the K334A mutant, the catalytic residue Lys334 is substituted by Ala. In the 3G mutant, the _423_SGNTT_427_ motif is substituted by three Gly residues to remove the beta turn and disrupt the beta finger made from β5 and β6. As depicted in Supplementary Fig. S9D, the side chains of Asn425 and Thr427 are involved in NS1 dodecamerization. Unlike WT NS1_200-501, the 3G mutant produces two elution peaks on the size-exclusion column. The molecular weight of the 12.0-ml peak corresponds to that of NS1_200-501 hexamer, whereas the 16.0-ml peak corresponds to monomeric NS1_200-501. Using duplex DNA-1 as substrate (Supplementary Fig. S3A), we performed *in vitro* DNA unwinding assays (Supplementary Fig. S17D). Although no DNA unwinding activity is observed for the K334A mutant, WT NS1_200-501 can efficiently unwind the DNA. The DNA unwinding activity of WT NS1_200-501 is comparable to that of WT NS1_2-570 (Fig. 1B). The hexameric 3G mutant can also efficiently unwind the DNA. Compared to WT protein, the DNA unwinding activity of the 3G mutant is even higher at low protein concentrations (0.1–1.0 μM). Our NS1_2-570/ssDNA/AMPPNP structure (Fig. 3A and B) clearly confirmed that NS1 functions as hexamer in DNA unwinding, which is identical to many other SF3 family helicases [40, 41]. We believed that the observed dodecamer of NS1 might be an artifact; the missing ZnD domain (Fig. 1A) might play certain role in dissociation of the two hexamers and enhancing the DNA unwinding activity of NS1.

### The unwinding activity of NS1 is dispensable for DNA cleavage

**Figure 6.**
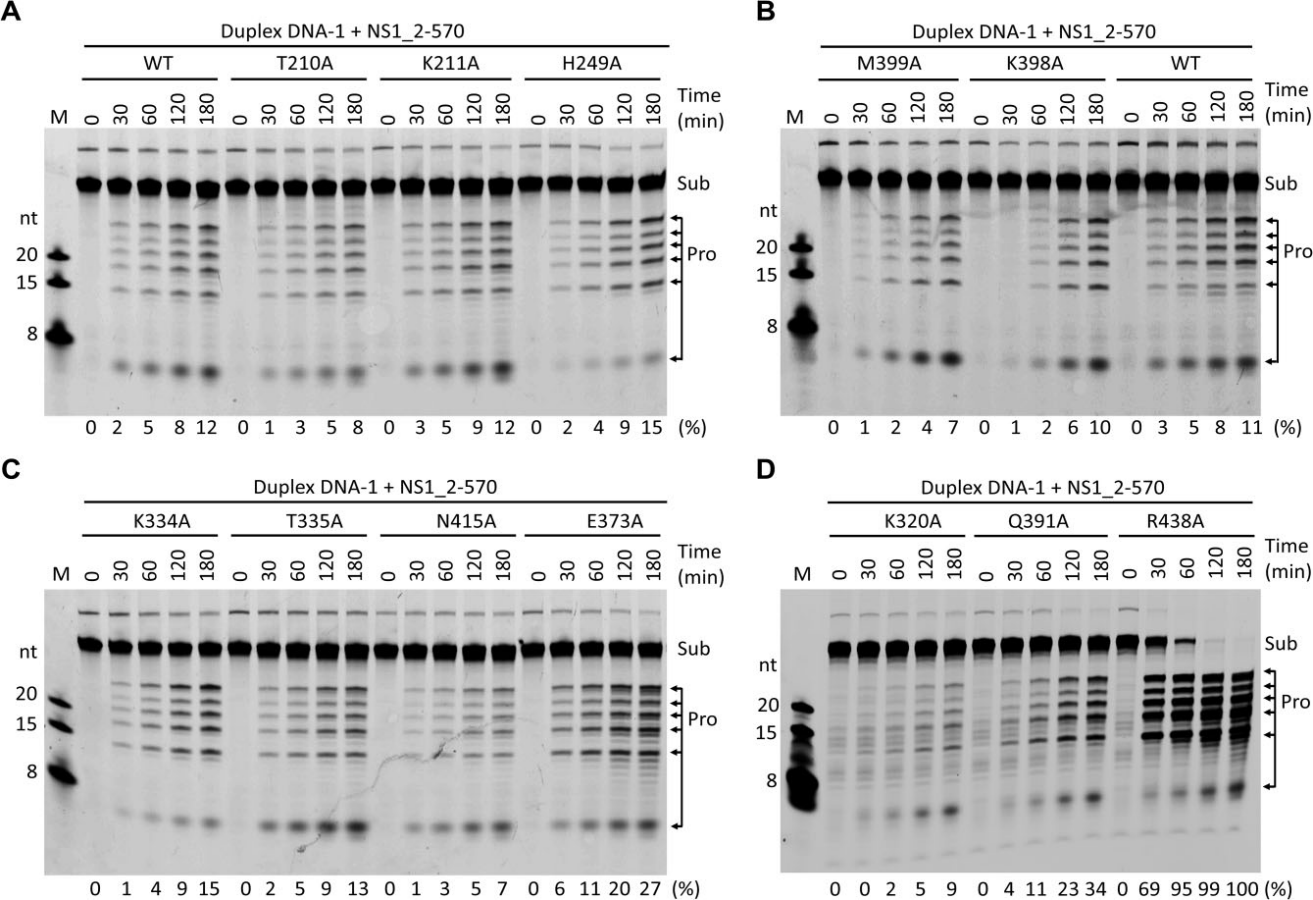
*In vitro* dsDNA cleavage assays. (**A**) Duplex DNA-1 cleavage by WT NS1_2-570 and mutants with the dsDNA-interacting residues mutated. (**B**) Duplex DNA-1 cleavage by WT NS1_2-570 and mutants with the ssDNA-interacting residues mutated. (**C**, **D**) Duplex DNA-1 cleavage by NS1_2-570 mutants with the ATP-interacting residues mutated. The substrate cleavage percentage (%) is shown at the bottom of the gels. Experiments were repeated independently three times with similar results.

Previous studies showed that the Nuc domain of NS1 can cleave ssDNA [21], but the natural targets encountered by NS1 are in the double-stranded form. To investigate whether NS1 can cleave dsDNA, we performed *in vitro* cleavage assays using the duplex DNA-1 as substrates. Duplex DNA-1 is composed of 48 base pairs, containing one predicted TRS site (5′-ACC-3′) and three NSBE motifs, NSBE1–NSBE3 (Supplementary Fig. S3A). As depicted in Fig. 6A, WT NS1_2-570 can cleave duplex DNA-1. Instead of single position, the DNA is cleaved by NS1_2-570 at multiple sites. Like WT NS1_2-570, the three mutants with dsDNA-interacting residues substituted by Ala, T210A, K211A, and H249A, can also cleave duplex DNA-1. The cleavage efficiency and product pattern of the three mutants are similar to that of WT protein.

Our *in vitro* assay results confirmed that many ssDNA-binding or ATP-binding residues play important roles in dsDNA unwinding by NS1 (Figs 2E and 4E). To clarify whether DNA unwinding is required for DNA cleavage by NS1, the K320A, K334A, T335A, E373A, Q391A, K398A, M399A, N415A, and R438A mutants of NS1_2-570 are utilized in the *in vitro* cleavage assays. As depicted in Fig. 6B–D, all the nine mutant proteins can cleave the duplex DNA-1. Although the DNA cleavage activities are slightly weakened for the K320A and K398A mutants, no obvious reduction is observed for the K334A, T335A, E373A, Q391A, M399A, or N415A mutants. Compared to WT NS1_2-570, the DNA cleavage activity of the R438A mutant is even higher (Fig. 6D). Taken together, these observations suggested that the DNA unwinding activity is dispensable for dsDNA cleavage by NS1.

### DNA cleavage by NS1 is negatively regulated by ATP binding or DNA unwinding

**Figure 7.**
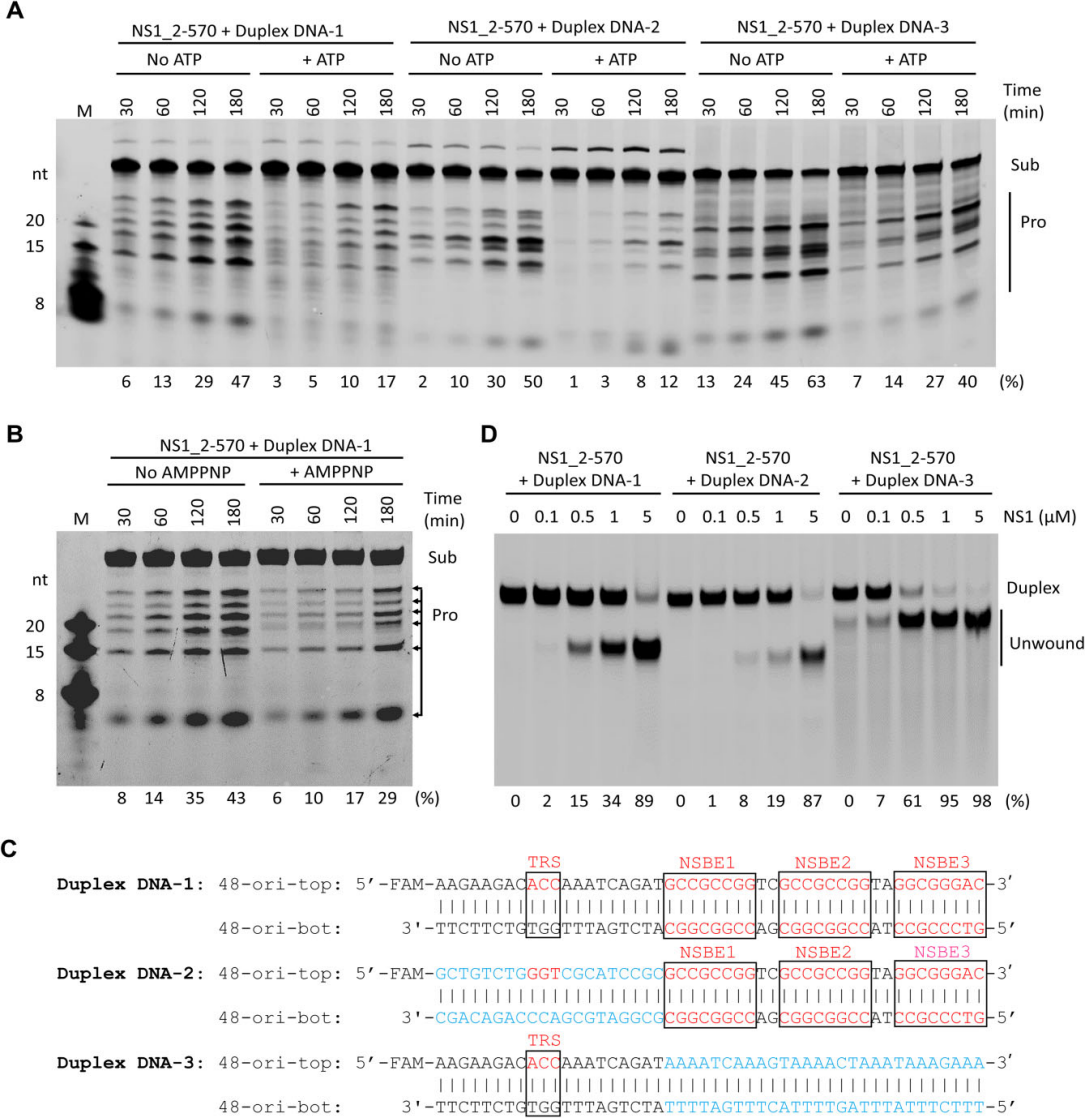
Correlation between DNA unwinding and DNA cleavage activities of NS1. (**A**) Comparison of *in vitro* dsDNA cleavage activity of NS1_2-570 in the presence or absence of ATP. (**B**) Comparison of *in vitro* duplex DNA-1 cleavage activity of NS1_2-570 in the presence or absence of AMPPNP. (**C**) Sequences of duplex DNA-1, duplex DNA-2, and duplex DNA-3. (**D**) *In vitro* dsDNA unwinding by NS1_2-570. The substrate cleavage percentage (%) is shown at the bottom of the gels. Experiments were repeated independently three times with similar results.

Arg438 is involved in AMPPNP binding in the NS1_2-570/ssDNA/AMPPNP structure (Fig. 4D); substitution of Arg438 with Ala significantly lowered the DNA unwinding activity of NS1_2-570 (Fig. 4E). However, compared to WT protein, the DNA cleavage activity of the R438A mutant is much higher (Fig. 6D). To better clarify the relationship between the DNA unwinding and DNA cleavage activities of NS1, we performed more *in vitro* assays. Compared to the reaction with ATP, NS1_2-570 shows higher duplex DNA-1 cleavage activity in the absence of ATP (Fig. 7A). Besides ATP, we also analyzed the DNA cleavage activity of NS1 in the presence or absence of AMPPNP. Although AMPPNP cannot be hydrolyzed by NS1, it can mimic ATP in binding and causing large conformational changes of NS1 (Fig. 4A–D). Compared to the reaction with AMPPNP, higher DNA cleavage activity was observed for NS1 in the absence of AMPPNP (Fig. 7B). In addition to DNA unwinding, these observations indicated that the conformational changes associated with ATP binding also have inhibitory effects on DNA cleavage by NS1.

The Ori of B19V contains one TRS and four NSBE motifs, which are rich in G-C base pairs [22]. To investigate whether these motifs affect Ori interaction with NS1, we synthesized two variants of duplex DNA-1, duplex DNA-2 and duplex DNA-3 (Fig. 7C). In duplex DNA-2, the TRS motif (5′-ACC-3′) was replaced by 5′-GGT-3′ and multiple G-C pairs were introduced into the flanking regions. In duplex DNA-3, many A-T base pairs are introduced into the NSBE motifs. As depicted in Fig. 7D, NS1_2-570 can unwind duplex DNA-2, but the unwinding efficiency is weaker than that of duplex DNA-1. Compared to both duplex DNA-1 and duplex DNA-2, the unwinding efficiency of duplex DNA-3 is higher, likely due to the presence of multiple A-T base pairs within the NSBE regions.

Like duplex DNA-1, the two variants are also used in the *in vitro* cleavage assays. As depicted in Fig. 7A, NS1_2-570 can cleave duplex DNA-2, and the cleavage efficiency is comparable to that of duplex DNA-1. Duplex DNA-3 can be cleaved by NS1_2-570, but the pattern of the products is very different from that of duplex DNA-2. Compared to the reactions without ATP, the cleavage efficiencies of the two variants are also weakened in the presence of ATP, which further confirmed that DNA cleavage by NS1 is negatively regulated by DNA unwinding.

The above assay results (Fig. 6A and B) clearly suggested that NS1 can cleave dsDNA. To gain more insights into dsDNA cleavage by NS1, we performed further *in vitro* assays using duplex DNA-1 and duplex DNA-4 as substrates (Supplementary Fig. S19A). The duplex DNA-4 is FAM-labeled at the 5′-end of the top strand. Compared to duplex DNA-1, duplex DNA-4 is 10 bp longer. Instead of the top strand, we also designed one duplex DNA-1 with FAM-labeled at the 5′-end of the bottom strand. As depicted in Supplementary Fig. S19B, both duplex DNA-1 strands can be cleaved by NS1. Compared to the top strand-labeled duplex DNA-1, cleavage of duplex DNA-4 produced one additional product; the patterns of other products are identical for the two DNAs, suggesting that the sequence of the DNA plays certain role in the cleavage site selection by NS1.

### DNA cleavage by NS1 requires the assistance of the 501–522 region

**Figure 8.**
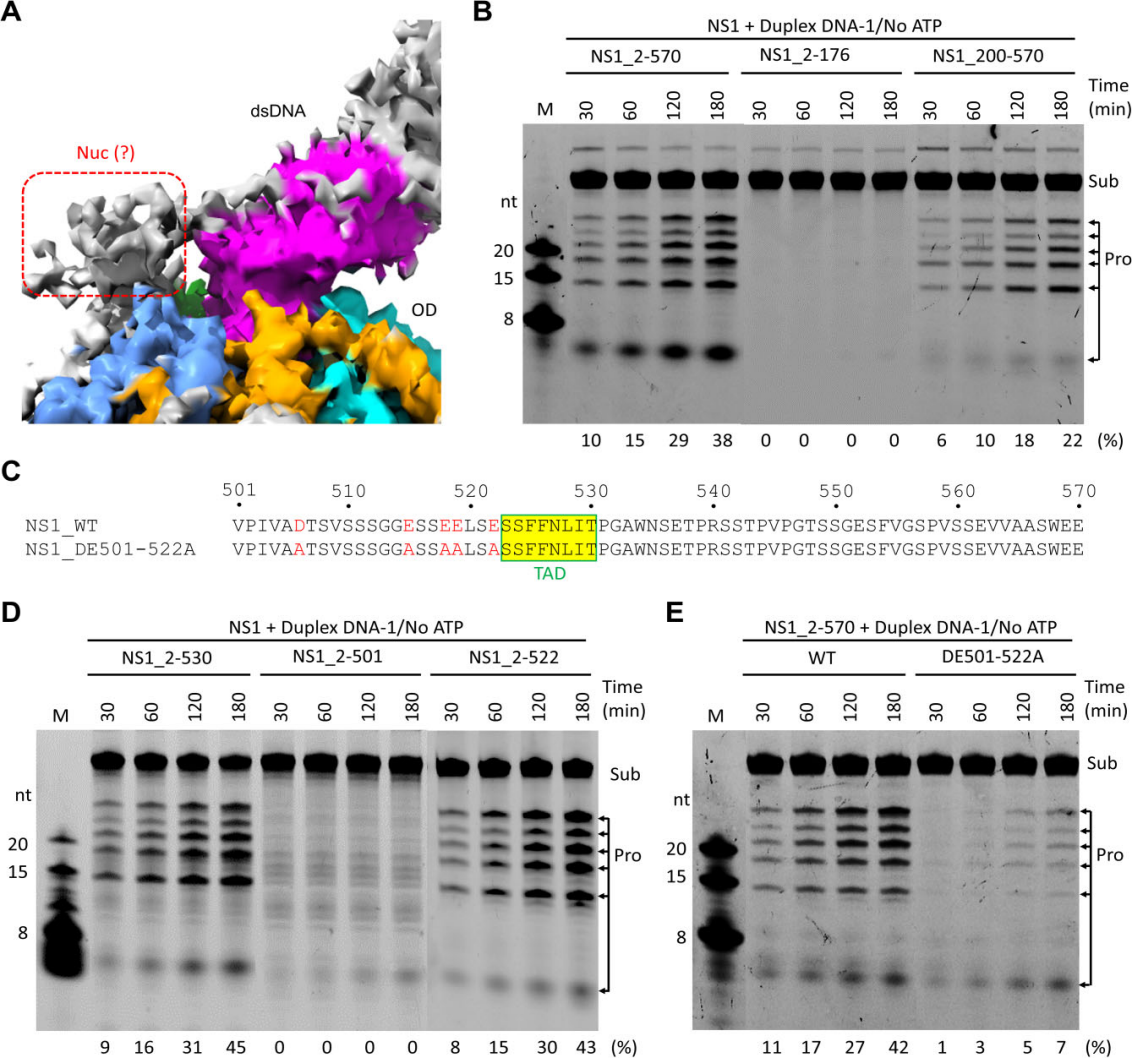
Identification of regions important for the DNA cleavage activities of NS1. (**A**) The close-up view showing the density maps for the potential NS1_Nuc domain observed in the NS1_2-570/dsDNA/AMPPNP structure. (**B**) Comparison of *in vitro* duplex DNA-1 cleavage activity of NS1_2-570, NS1_2-176, and NS1_200-570. (**C**) Sequences of the 501–570 region in WT NS1_2-570 and the DE501-522A mutant protein. (**D**) Comparison of duplex DNA-1 cleavage activity of NS1_2-501, NS1_2-522, and NS1_2-530 mutants. (**E**) Comparison of duplex DNA-1 cleavage activity of WT and the DE501-522A mutant proteins of NS1_2-570. All assays were done in the absence of ATP. The substrate cleavage percentage (%) is shown at the bottom of the gels. Experiments were repeated independently three times with similar results.

Like the OD and SF3 HD domains, the Nuc domain is also present in the protein used in the dsDNA-bound structure. Next to dsDNA, some extra density could be observed (Fig. 8A). Although not sufficient for model building, the density is most likely produced by the Nuc domain. Our structure and *in vitro* assay results (Supplementary Fig. S17C and D) confirmed that the OD and SF3 HD domains are sufficient for dsDNA unwinding by NS1. To clarify which domains are required for DNA cleavage by NS1, we purified two truncated proteins, NS1_2-176 and NS1_200-570 (Supplementary Fig. S20), and performed *in vitro* cleavage assays using duplex DNA-1 as substrates. As depicted in Fig. 8B, NS1_2-176 has no obvious DNA cleavage activity. NS1_200-570 can cleave DNA, but its efficiency is weaker than that of NS1_2-570, suggesting that efficient DNA cleavage requires the corporation of multiple regions of NS1.

The 501–570 regions (Fig. 8C) are present in the proteins used in the cryo-EM studies, but they were not observed in any NS1 structures. In contrast to NS1_2-570, no obvious DNA cleavage activity could be observed for the NS1_2-501 protein (Fig. 8D and Supplementary Fig. S20), suggesting that the 501–570 region participates in DNA cleavage by NS1. To further clarify which part of 501–570 involves in DNA cleavage, two additional proteins were purified, NS1_2-530 and NS1_2-522 (Supplementary Fig. S20). As depicted in Fig. 8D, both NS1_2-530 and NS1_2-522 can cleave duplex DNA-1; the cleavage efficiencies of the two proteins are similar, suggesting that the TAD domain (aa 523–530) is not essential for DNA cleavage.

Sequence analysis revealed that the 501–522 region is rich in acidic residues (Fig. 8C). To investigate the potential function of these residues, we constructed one NS1_2-570 mutant protein, DE501-522A, in which all acidic residues within the 501–522 region are substituted with Ala (Fig. 8C and Supplementary Fig. S20). As depicted in Fig. 8D, DE501–522A can cleave duplex DNA-1. However, when compared to that of WT NS1_2-570, the DNA cleavage activity of DE501–522A is much weaker, suggesting that the acidic residues within the 501–522 region participates in DNA cleavage by NS1.

## Discussion

Since infection of B19V could cause various diseases in humans, it has been extensively studied in the past; however, no efficient anti-B19V drugs have been development to date. Seasonal outbreaks of B19V normally occur in the late winter and early spring [47], but unusual epidemics of B19V in other seasons has been reported in more and more countries in recent years [48–50], which turned B19V into a global concern. As the main replication protein of B19V, NS1 is composed of multiple domains (Fig. 1A). The structural basis underlying the function of NS1 is largely unknown, due to the difficulty in obtaining high-quality proteins. To the best of our knowledge, our *in vitro* assays confirmed the DNA unwinding activity of NS1 for the first time. NS1 is capable of unwinding various types of DNAs (Fig. 1B and C), which sets NS1 apart from many other SF3 family helicases, such as Mpox E5 protein and papillomavirus E1 helicase that preferentially unwind DNA with 3′ overhang [39].

NS1 shares similar domain architecture with the Rep72 protein from AAV (Fig. 1A). Like B19V, AAV also belongs to the *Parvoviridae* family. Due to its great potential in gene therapy, AAV has been extensively utilized and considered as an excellent model for studying biological processes in parvovirus. It was found that Rep72 and Rep68, which are similar to NS1_2-570 in domain architecture, are involved in all DNA transactions required for the life cycle of AAV, such as DNA replication, site-specific integration, and genome packaging [51]. The smaller Rep proteins, Rep52 and Rep40, mainly function in the package of the genome [52]. Due to the lack of cofactor and/or substrate-bound structures in the catalytic forms, the basis underlying the function of the Rep proteins is poorly understood. B19V NS1 and AAV Rep proteins share significant sequence and structural similarity within their Nuc, OD, and SF3 HD domains (Supplementary Figs S21 and S22). The cofactor ATP binding and hydrolysis mechanism revealed by the NS1_2-570–ssDNA–AMPPNP structure should be conserved in AAV Rep and helicase proteins from other parvoviruses.

To meet with their function in multiple biological processes, AAV Rep72 and Rep68 could assemble into either hexamer or heptamer [53]. As the inner diameter is large enough, no significant conformational change is required for dsDNA to pass through the central ring of the heptameric Rep structure. The assembly of the hexameric Rep structure is very similar to that of NS1_2-570 in the ssDNA-bound structure (Supplementary Fig. S23A). Although the detailed orientations of the SF3 HD domains are not identical (Supplementary Fig. S23B), the ring formed by the OD domains is very similar in NS1_2-570 and the hexameric Rep structures, suggesting that hexamer is the functional form of Rep72 and Rep68 for DNA unwinding. An ssDNA-bound hexameric Rep68 structure was previously reported [53], but the DNA was not well modeled due to the limitation of resolution. Instead of extended conformation, structural superposition suggested that ssDNA bound by Rep68 SF3 HD domain should also adopt the B-form-like conformation (Supplementary Fig. S23C).

Besides the OD and SF3 HD domains, the Nuc domain is also present in NS1_2-570. As indicated by the extra density in the dsDNA-bound structure, one Nuc domain resides near the bound dsDNA (Fig. 8A). Superposition the isolated NS1_Nuc structure (PDB_ID: 7Y56) with the dsDNA-bound Rep68_Nuc structure (PDB_ID: 4ZQ9) [54] suggests that NS1_Nuc may also use two DNA-interacting loops, DIL-1 and DIL-2, in dsDNA binding. Although the Nuc domain is not essential for dsDNA cleavage by NS1, its truncation lowers the dsDNA cleavage activity of the protein (Fig. 8D). Surprisingly, our *in vitro* assay results revealed that the acidic residues within the 501–522 region also participates in dsDNA cleavage by NS1 (Fig. 8E). In the future, further structural studies are needed to clarify how the Nuc domain and the 501–522 region involve in dsDNA cleavage.

Our *in vitro* assays confirmed that NS1 possesses both DNA unwinding (Fig. 1B) and DNA cleavage activities (Fig. 6). The SF3 HD domain plays essential role in DNA unwinding by NS1. In addition to NS1, the SF3 HD is also present in many other multi-domain proteins, such as the large tumor antigen protein of simian virus 40 [42], the C962R protein of African swine fever virus [55], and the NrS-1 protein from the deep-sea vent phage [56]. The helicase domains of these proteins are normally evolved to enhance the catalytic activities of other domains. However, instead of enhancing, DNA unwinding by the SF3 HD domain has an inhibitory effect on DNA cleavage by NS1 (Fig. 7A and B). This unique property differs NS1 from other helicase-containing multi-domain proteins. The individual domain is likely co-evolved to fulfill the function of NS1 *in vivo*. The Nuc domain is required for DNA binding and cleavage at the TRS site of the target DNA in the single-strand form [21]. The OD domain is required for the hexamerization, which is essential for the DNA unwinding activity of the SF3 HD domain. Although the detailed basis is not clear at present, the 501–522 region and the 523–530 region are required for dsDNA cleavage (Fig. 8E) and the protomer transactivation activity of NS1 [24], respectively.

Replication of the B19V genome follows the “rolling hairpin” mechanism [18]. The DNA cleavage and unwinding activities of NS1 are likely required during different stages of the replication. Cleavage by NS1 at the hairpin structure of the genome generates free 3′-OH group, which will be utilized by the host polymerase for DNA synthesis. Being able to unwind various types of DNAs (Fig. 1B), NS1 may facilitate the strand separation and displacement steps of the replication intermediates. The dsDNA cleavage activity of NS1 is negatively regulated by DNA unwinding (Fig. 7A and B). Although it remains to be further investigated, this regulation may prevent NS1 from nonspecific cleavage, such as the cleavage at other dsDNA regions of the B19V genome replication intermediate. NS1 contains one ZnD domain at its C-terminus. In the future, it is worth investigating whether this ZnD domain can enhance the cleavage efficiency and specificity of NS1.

In conclusion, we performed extensive structural and biochemical studies on NS1, which revealed the detailed basis for DNA unwinding and the regions required for DNA cleavage by NS1. Besides B19V, this study also advances our understanding on the function and catalytic mechanisms of AAV Rep72 and Rep68 and helicases from other parvoviruses.

## Supplementary Material

gkaf562_Supplemental_File

## Data Availability

Structural coordinates have been deposited in the Protein Data Bank under accession codes 9KBG, 9KBH, 9KBI, and 9KBJ for the NS1_2-570/AMPPNP complex, the NS1_2-570/ssDNA/AMPPNP complex, the NS1_2-570/dsDNA/AMPPNP complex, and the NS1_200-501/AMPPNP complex, respectively.
