# Supplementary material for: Structural and functional studies of the main replication protein NS1 of human parvovirus B19
Source: Nucleic Acids Res. 2025 Jun 26;53(12):gkaf562. doi: 10.1093/nar/gkaf562 (PMC12199161; doi:10.1093/nar/gkaf562)
Supplement: gkaf562_Supplemental_File [file gkaf562_supplemental_file.pdf]

Supplementary Information

For

Structural and functional studies of the main replication protein NS1 of human parvovirus B19

Yixi Zhang[1], Boming Fan[1], Yanqing Gao[1], Jie Yang[1], Weizhen Zhang[1], Shichen Su[1], Linxi Li[1], Huili Li[1], Zhaorong Luo[1], Guangli Tang[1], Chenxi Wang[1], Xueting Zhang[1], Hehua Liu[1], Jianhua Gan[1,*]

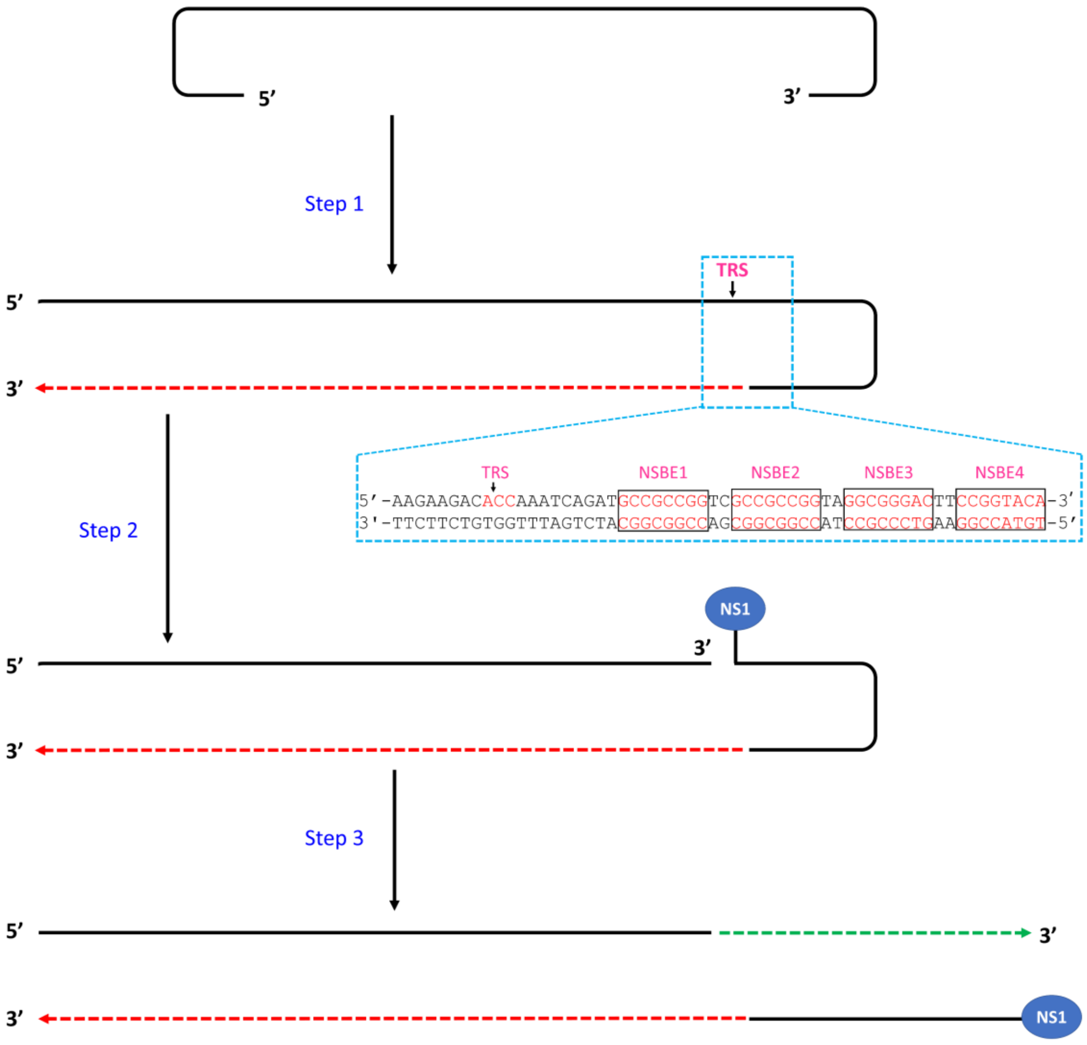

Fig. S1: Rolling-hairpin model of B19V replication. At step 1, the 3' end of one terminal hairpin of B19V DNA is extended by a cellular polymerase. At step 2, NS1 binds B19V DNA origin and cleaves at the terminal resolution site (TRS), generating a new 3'-end. At step 3, the new 3'-end is extended by polymerase, completing the synthesis of the DNA.

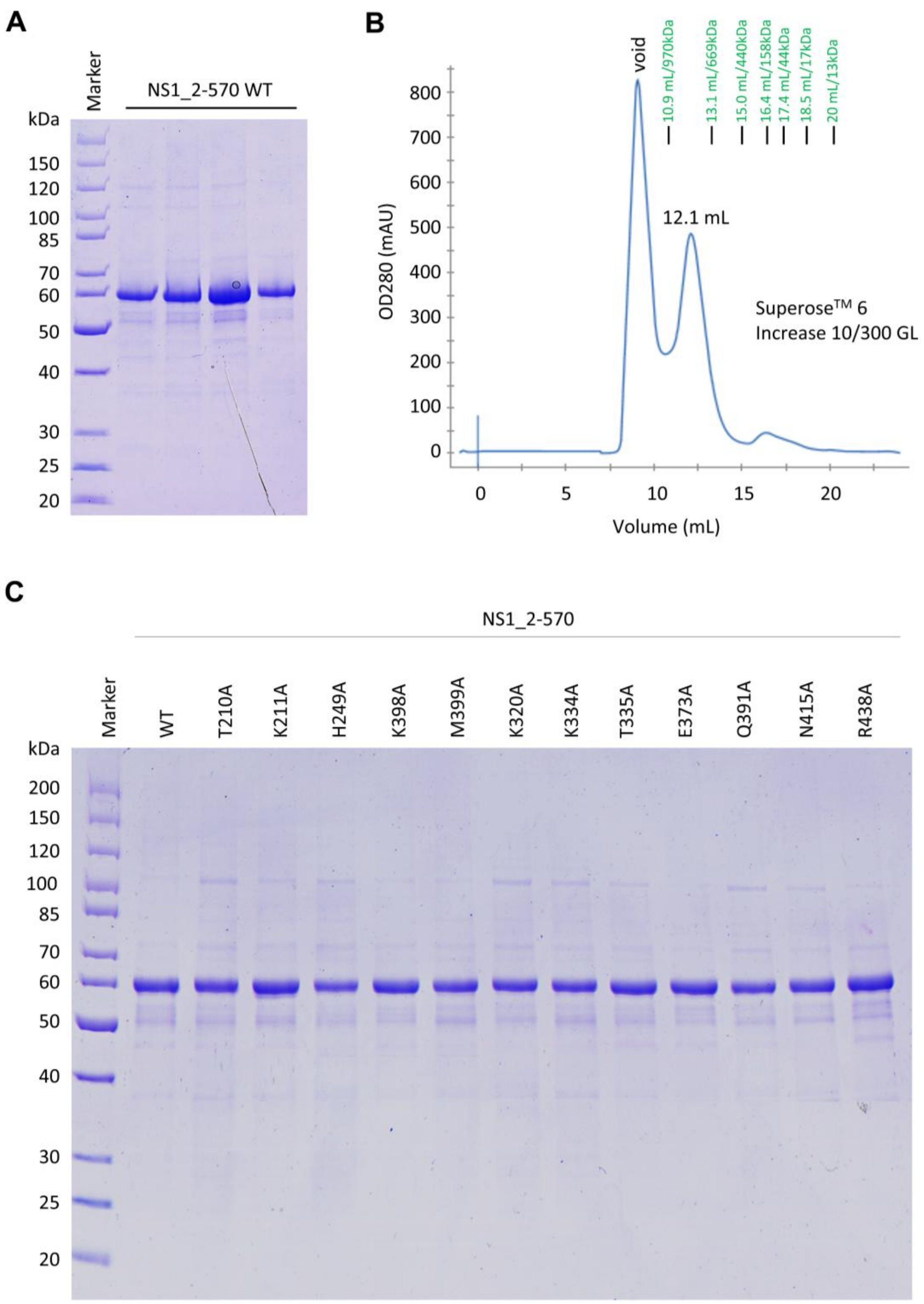

Fig. S2: SDS-page gel analysis and size-exclusion chromatography profile of NS1_2-570. (**A**) SDS-page gel analysis of WT NS1_2-570. (**B**) The size-exclusion chromatography profile of WT NS1_2-570. (**C**) SDS-page gel analysis of WT and mutant proteins of NS1_2-570.

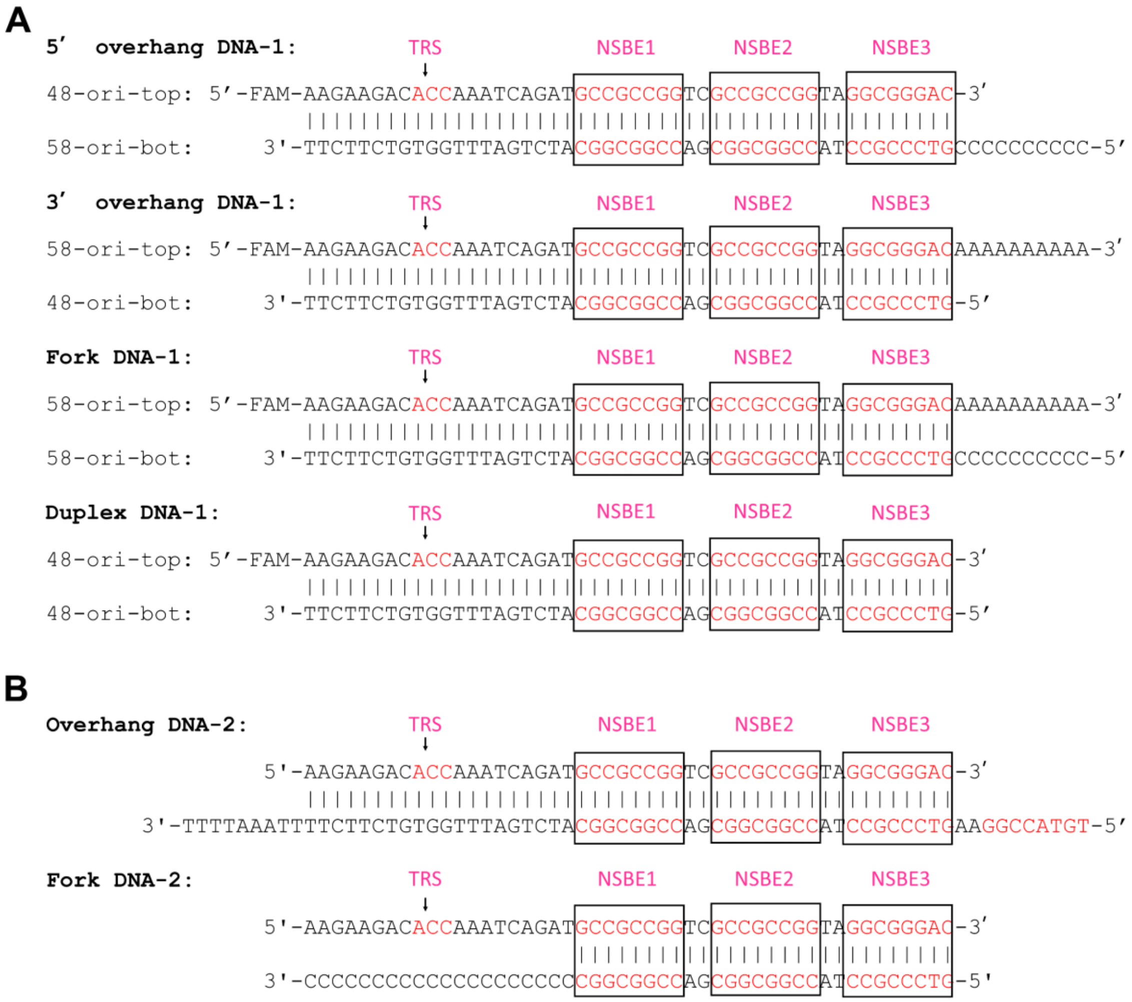

Fig. S3: Sequences of DNAs utilized in this study. (**A**) DNAs used in the *in vitro* unwinding or cleavage assays. (**B**) DNAs used in the structural studies of NS1_2-570. TRS stands for the terminal resolution site. NSBE1, NSBE2, and NSBE3 stand for the NS1-binding elements 1, 2, and 3, respectively.

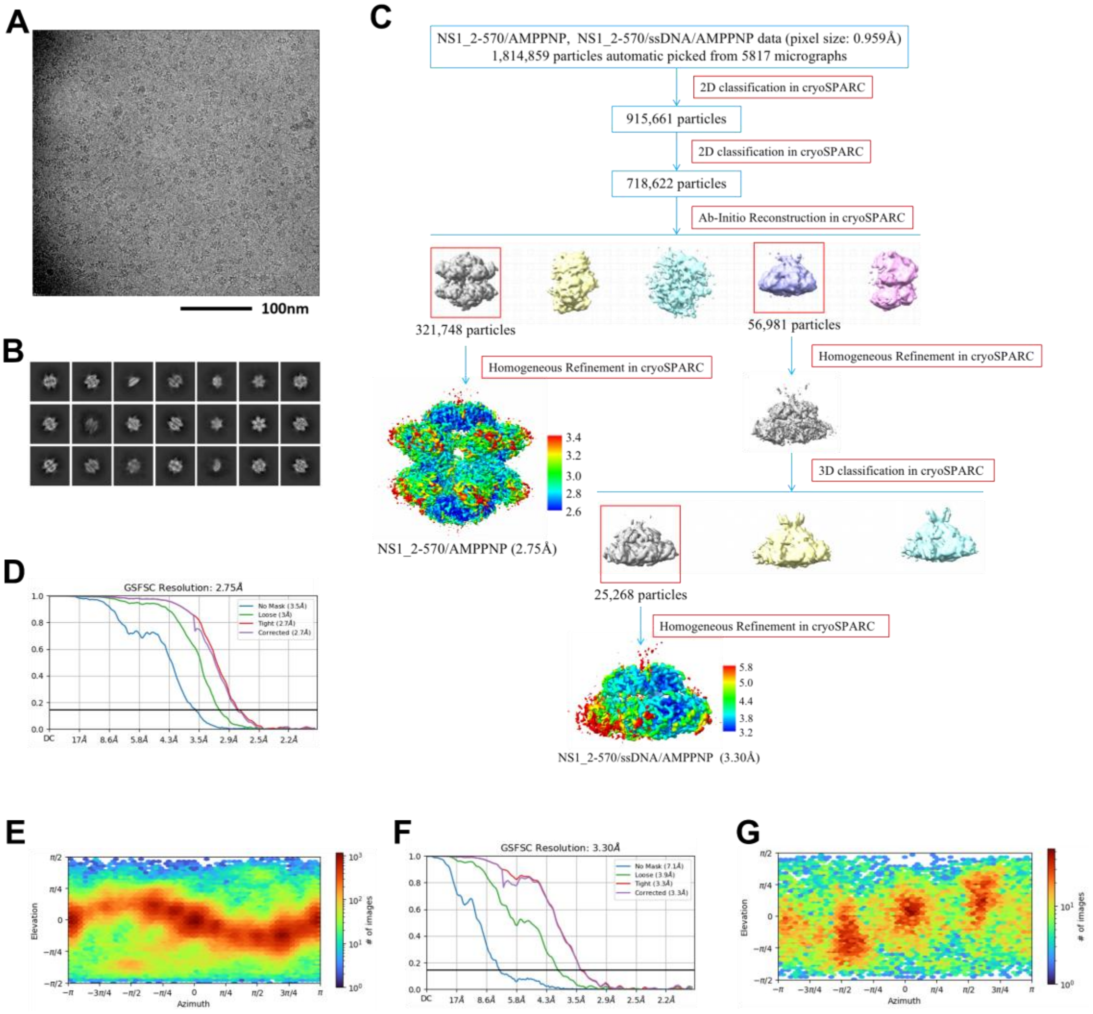

Fig. S4: Cryo-EM data processing of the NS1_2-570/AMPPNP and NS1_2-570/ssDNA/AMPPNP structures. (**A**) A representative cryo-EM image of the NS1_2-570/AMPPNP and NS1_2-570/ssDNA/AMPPNP structures. (**B**) Representative 2D classification images of the NS1_2-570/AMPPNP and NS1_2-570/ssDNA/AMPPNP structures. (**C**) Flowchart of cryo-EM data processing and final density map colored according to the local resolution. (**D, F**) Gold-standard Fourier shell correlation (GSFSC) of the final map of the NS1_2-570/AMPPNP and NS1_2-570/ssDNA/AMPPNP structures, respectively. (**E, G**) Particle orientation distribution of the NS1_2-570/AMPPNP and NS1_2-570/ssDNA/AMPPNP structures, respectively.

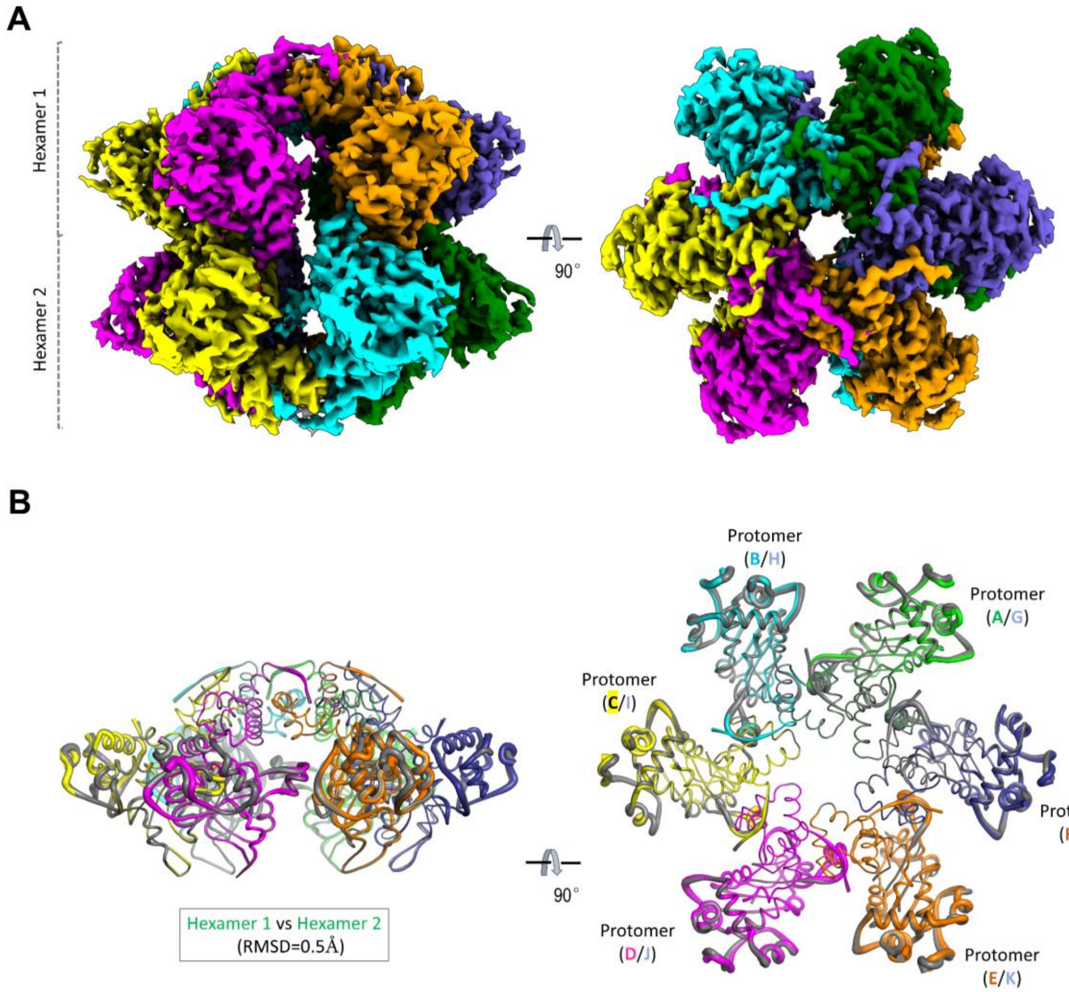

Fig. S5: The NS1_2-570/AMPPNP structures. (**A**) The final density maps of the NS1_2-570/AMPPNP structure. (**B**) Superposition of the two NS1_2-570 hexamers observed in the NS1_2-570/AMPPNP structure. In panel B, both hexamers are presented in sausage views based on the B factors. The NS1_2-570 protomers A to F are colored in green, cyan, yellow, magenta, orange, and blue in the upper hexamer, respectively. All the protomers in the lower hexamer are colored in gray.

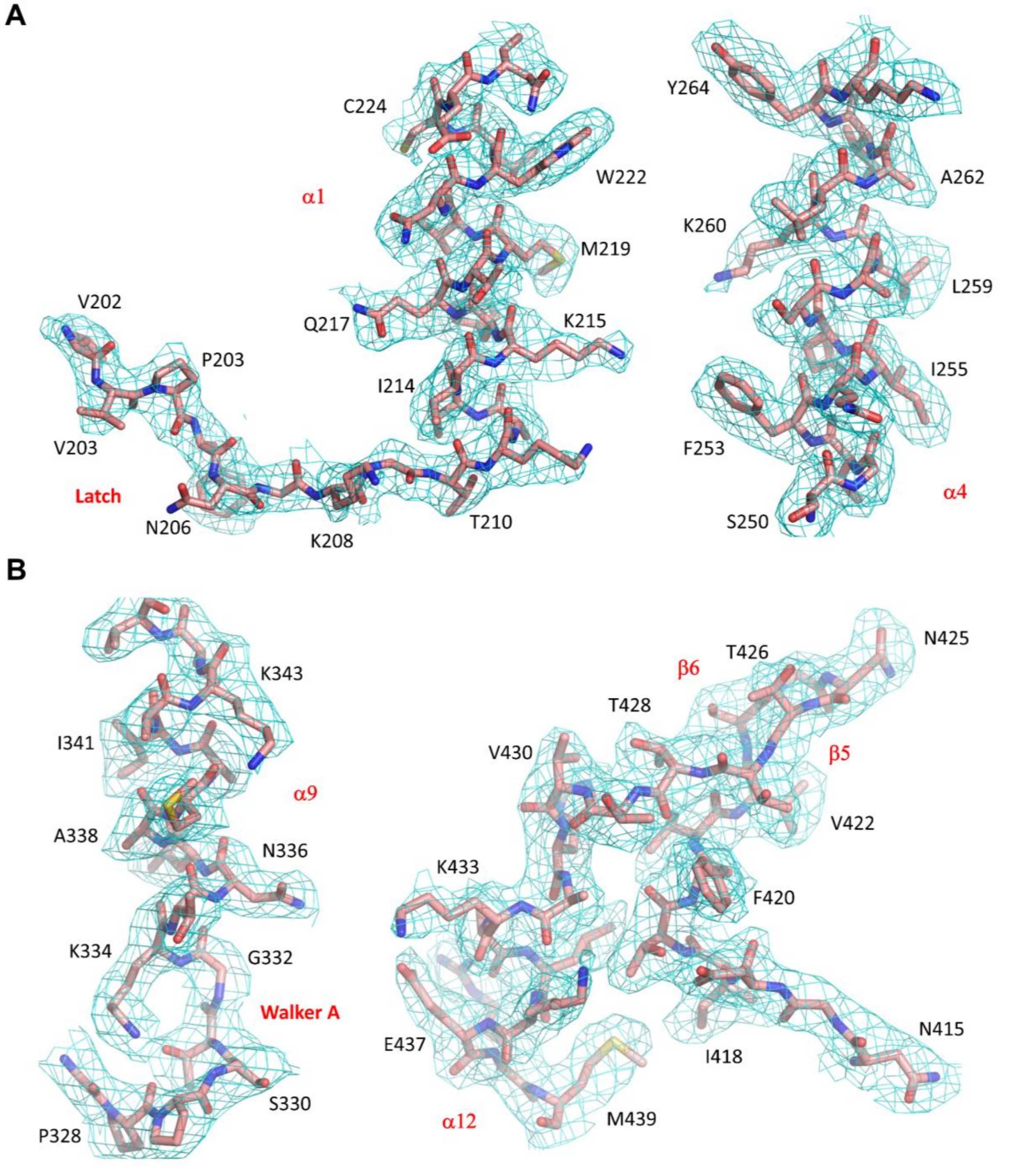

Fig. S6: Representative cryo-EM density maps of the NS1 protein. (**A**) The density maps for residues from the OD domain. (**B**) The density maps for residues from the SF3 HD domain.

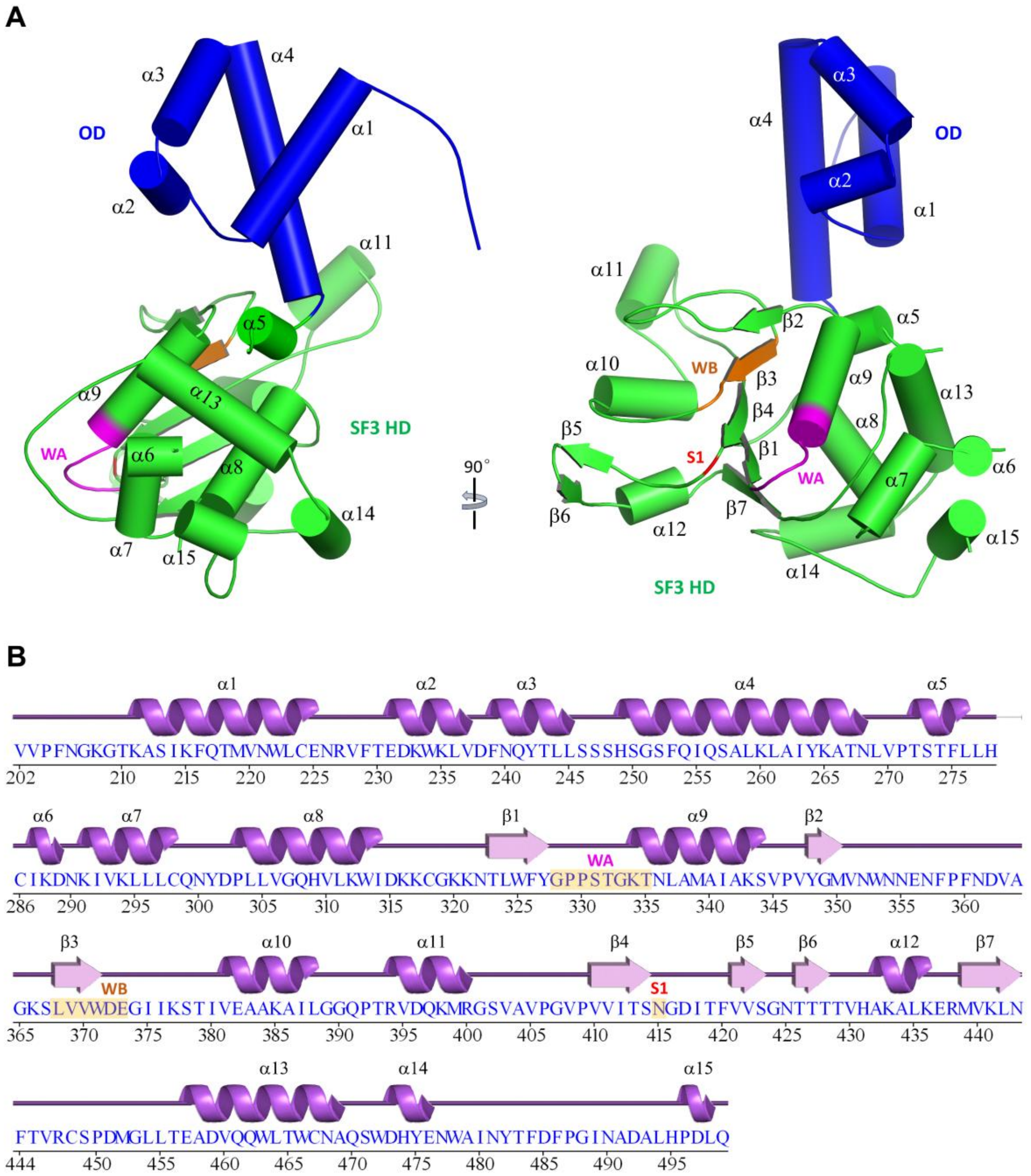

Fig. S7: Overall folding and secondary structures of NS1_2-570. (**A**) The cartoon presentation of the NS1_2-570 protein. The Nuc domain is disordered in the structure. (**B**) The secondary structures of the OD and SF3 HD domains of NS1. WA, WB, and S1 stand for the Walker A motif, Walker B motif, and sensor 1, respectively.

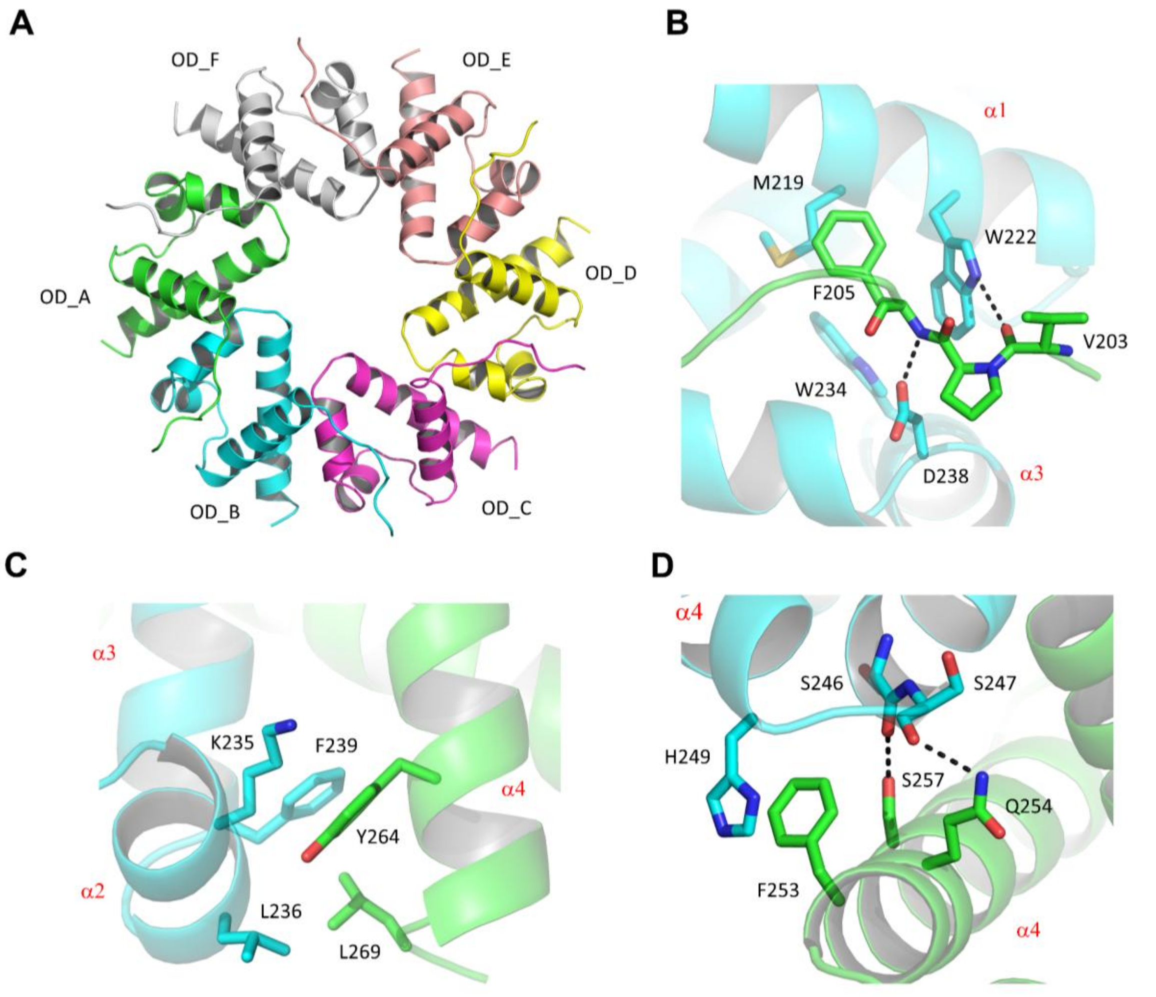

Fig. S8: Hexamerization of NS1_2-570. (**A**) The ring formed by the OD domain of NS1. (**B**) The interactions mediated by the N-terminal loop of the OD domain. (**C-D**) The interactions mediated by residues of the helices α2 and α4.

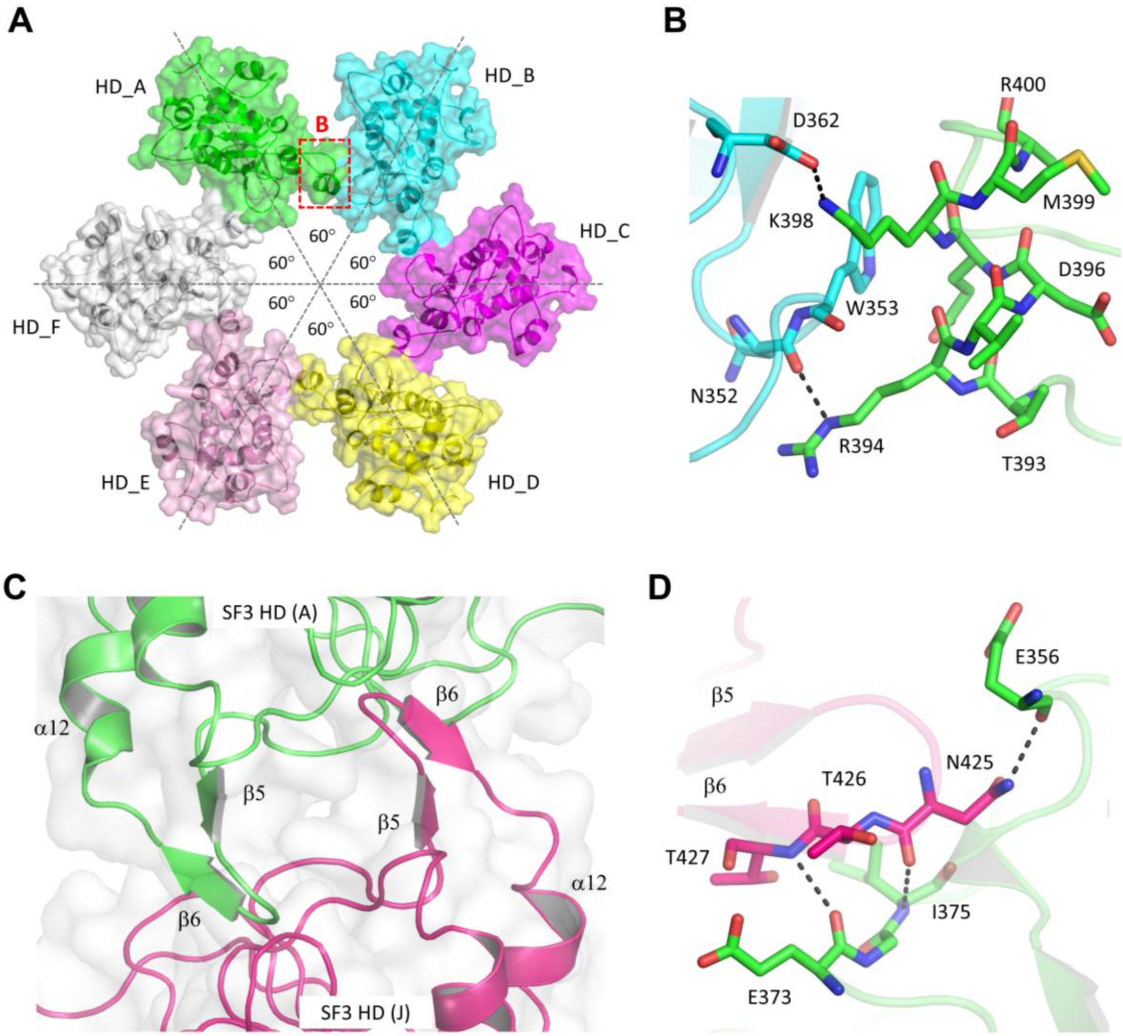

Fig. S9: Interactions mediated by the SF3 HD domains. (**A**) The ring formed by the SF3 HD domain of NS1. (**B**) The interactions mediated by the 393-400 region of the SF3 HD domain. (**C-D**) The interactions involved in NS1 dodecamerization.

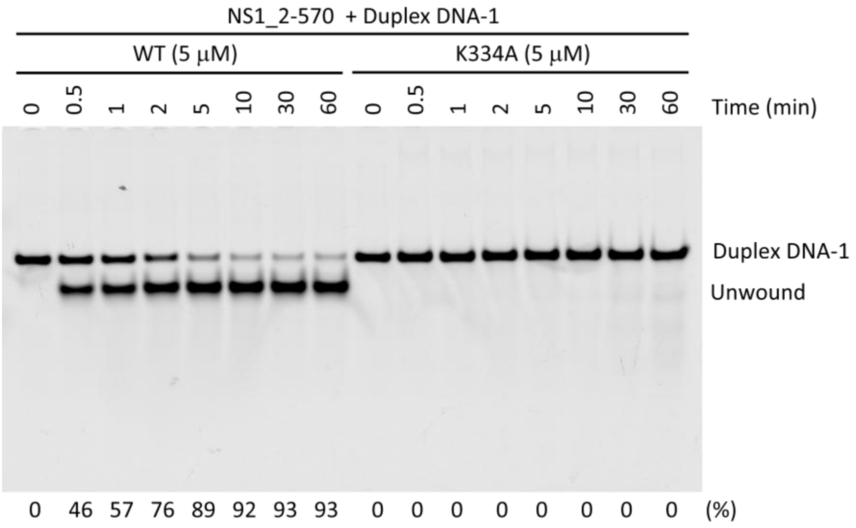

Fig. S10: Time course analysis of duplex DNA-1 unwinding by NS1_2-570 proteins. The substrate unwinding percentage (%) is shown at the bottom of the gels. Experiments were repeated independently three times with similar results.

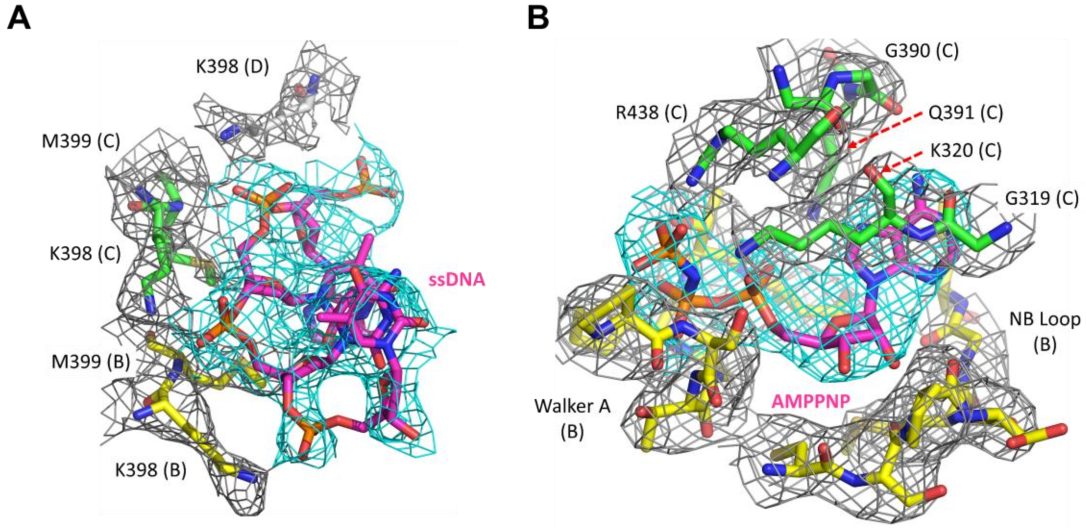

Fig. S11: Density maps observed in the NS1_2-570/ssDNA/AMPPNP structure. (**A**) Density maps of ssDNA and ssDNA-interacting residues. (**B**) Density maps of AMPPNP and AMPPNP-interacting residues.

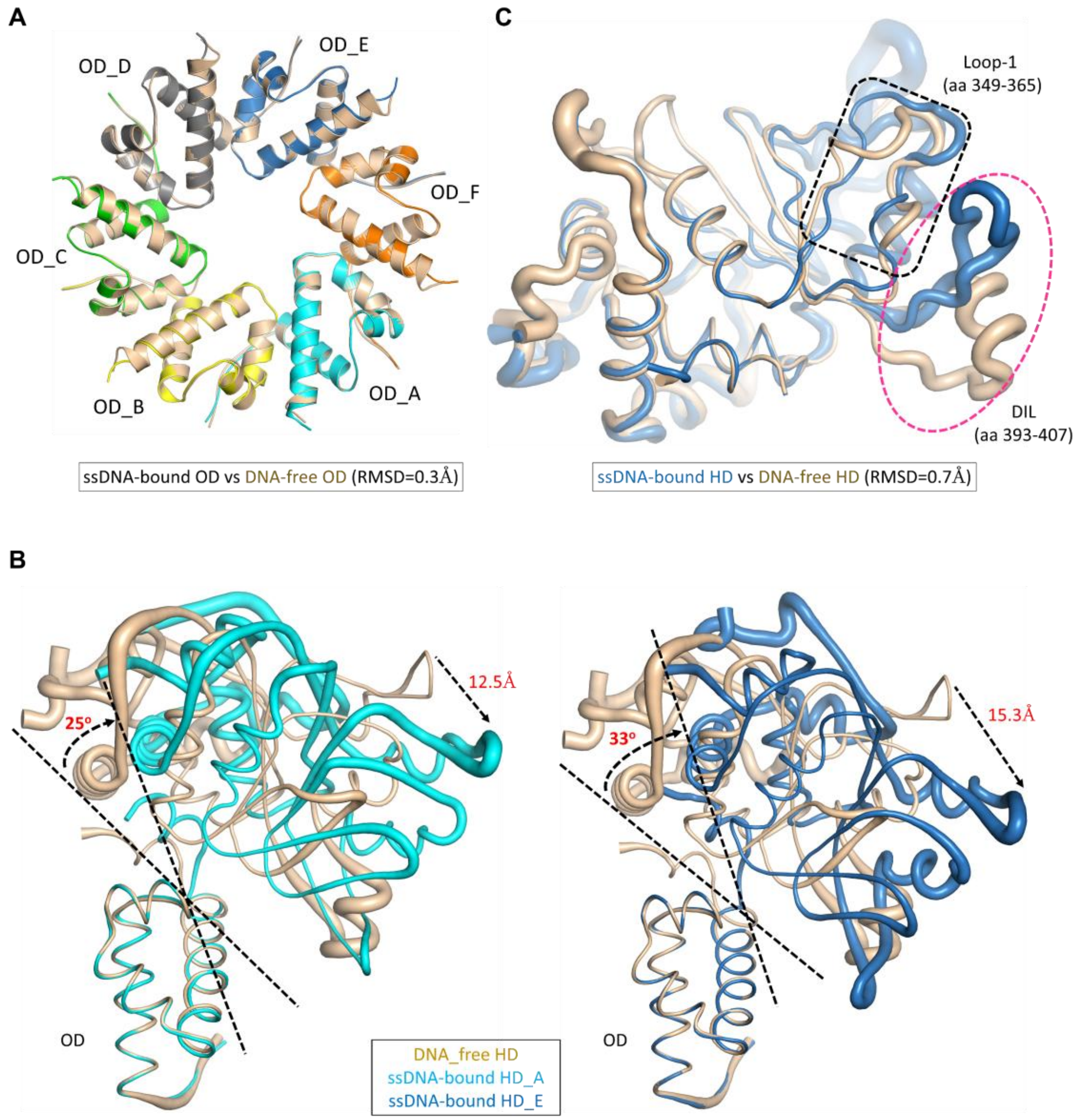

Fig. S12: Comparison of the DNA-free and ssDNA-bound NS1_2-570 structures. (**A**) Superposition of the rings formed by the OD domains. (**B**) Superposition showing the orientational changes between the OD and SF3 HD domains. (**C**) Superposition showing the local conformational changes of the SF3 HD domains. All protomers in the DNA-free structure are colored in pink, whereas the protomers A to F are colored in cyan, yellow, green, gray, blue, and orange in the ssDNA-bound structure. In panels **B** and **C**, both structures are presented in sausage views based on the B factors.

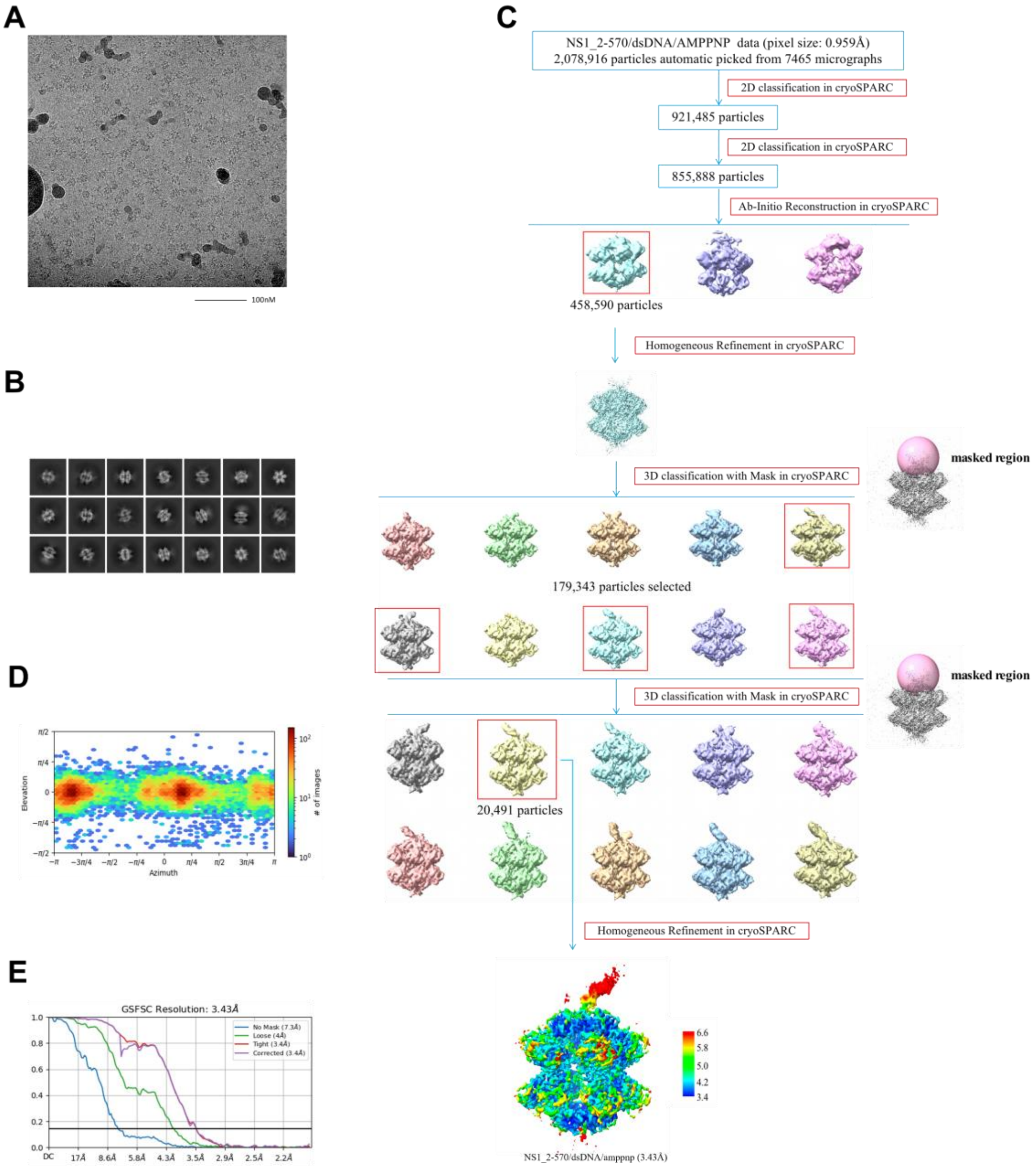

Fig. S13: Cryo-EM data processing of the NS1_2-570/dsDNA/AMPPNP structure. (**A**) A representative cryo-EM image of the NS1_2-570/dsDNA/AMPPNP structure. (**B**) Representative 2D classification images of the NS1_2-570/dsDNA/AMPPNP structure. (**C**) Flowchart of cryo-EM data processing and the final density map colored according to the local resolution. (**D**) Particle orientation distribution of the NS1_2-570/dsDNA/AMPPNP structure. (**E**) Gold-standard Fourier shell correlation (GSFSC) of the final map of the NS1_2-570/dsDNA/AMPPNP structure.

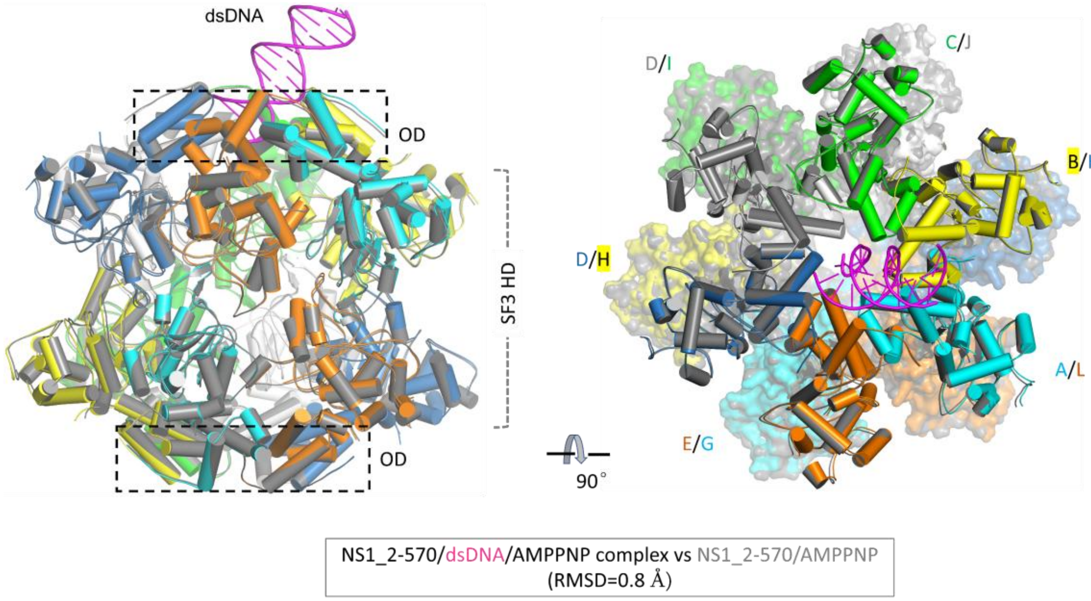

Fig. S14: Comparison of the DNA-free and dsDNA-bound NS1_2-570 structures. For the dsDNA-bound structure, the dsDNA is shown as cartoon in magenta. The protomers are colored in different colors. For the DNA-free structure, all the protomers are colored in gray.

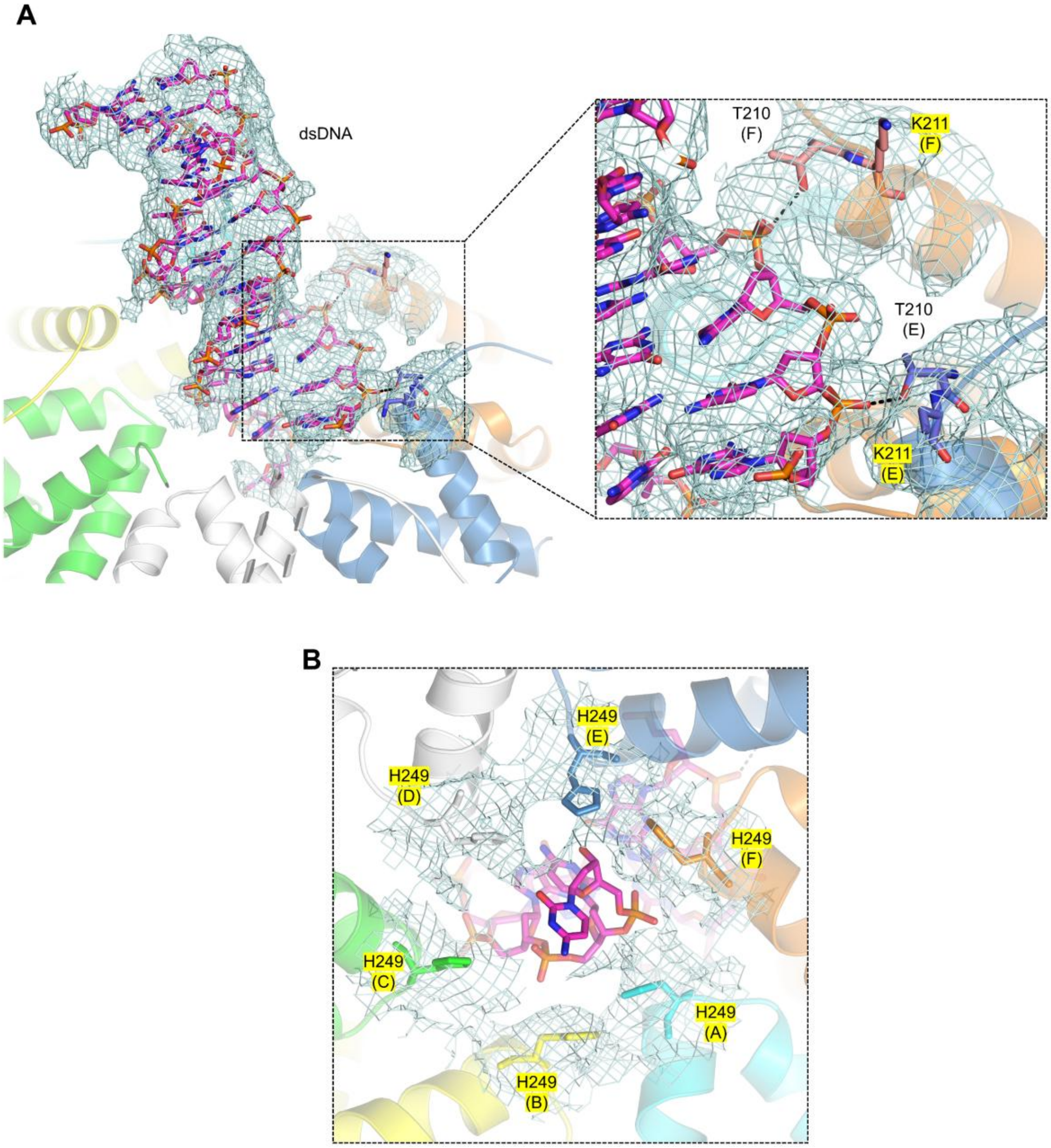

Fig. S15: Cryo-EM density maps of the dsDNA-bound NS1_2-570 structure. (**A**) Density maps of the dsDNA and the surrounding residues, Thr210 and Lys211. (**B**) Density maps of His249 located at the entry site of the DNA.

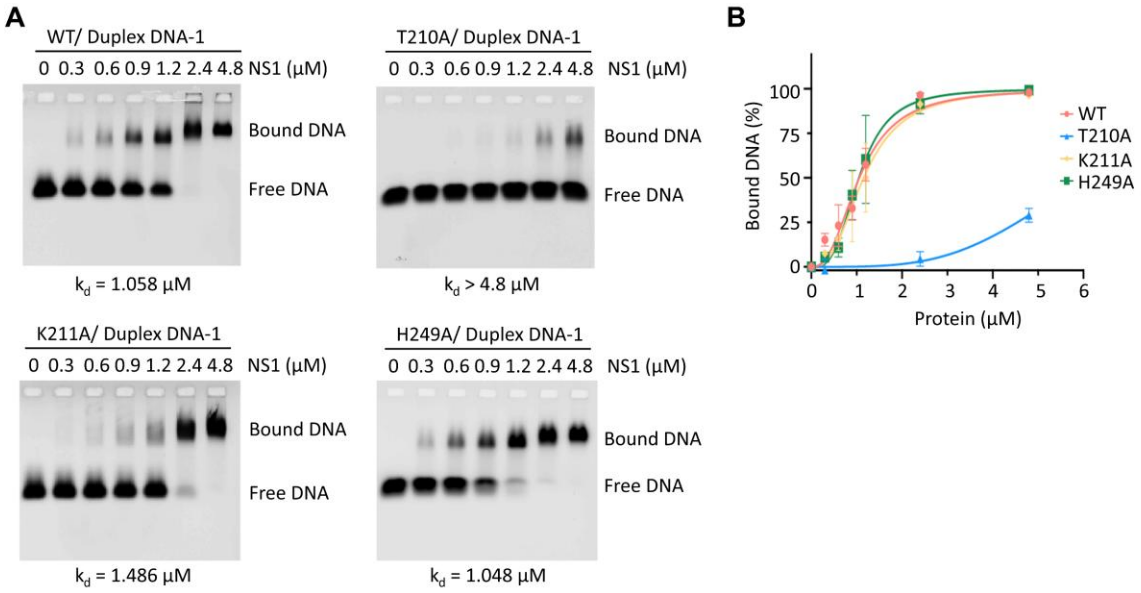

Fig. S16: Comparison of the dsDNA biding affinities of WT NS1_2-570 and three mutant proteins. Duplex DNA-1 was used as substrates in these EMSA assays. Experiments were repeated independently three times with similar results.

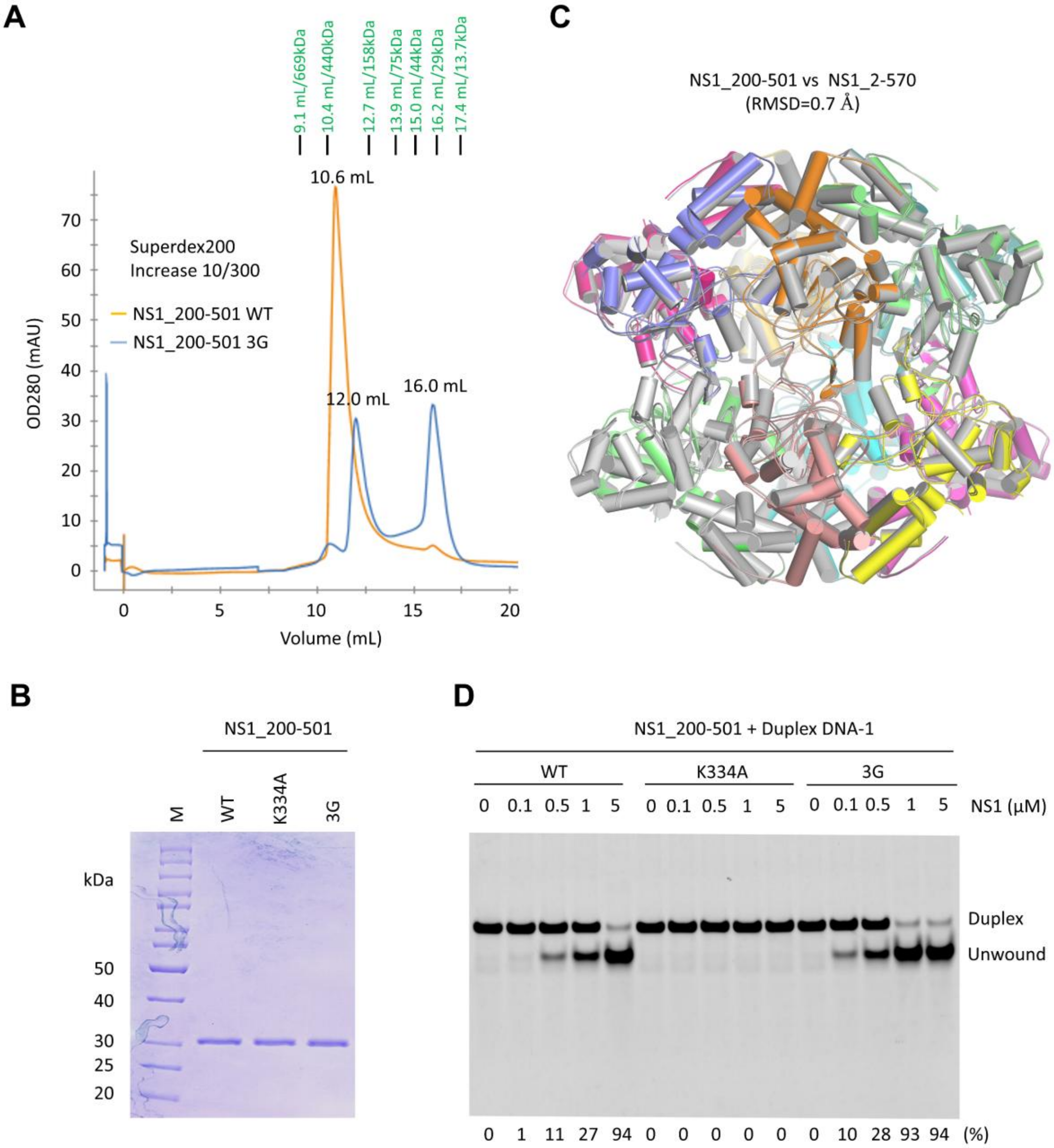

Fig. S17: Characterization of the 200-501 region of NS1. (**A**) Size-exclusion profiles of WT and 3G mutant of NS1_200-501. (**B**) SDS-page gel analysis of WT and mutants of NS1_200-501. (**C**) Superposition of the DNA-free NS1_200-501 and NS1_2-570 structures. The protomers in the NS1_200-501 structure are colored with different colors, whereas they are all colored in gray in the NS1_2-570 structure. (**D**) *In vitro* DNA unwinding catalyzed by WT and mutant proteins of NS1_200-501. Experiments were repeated independently three times with similar results.

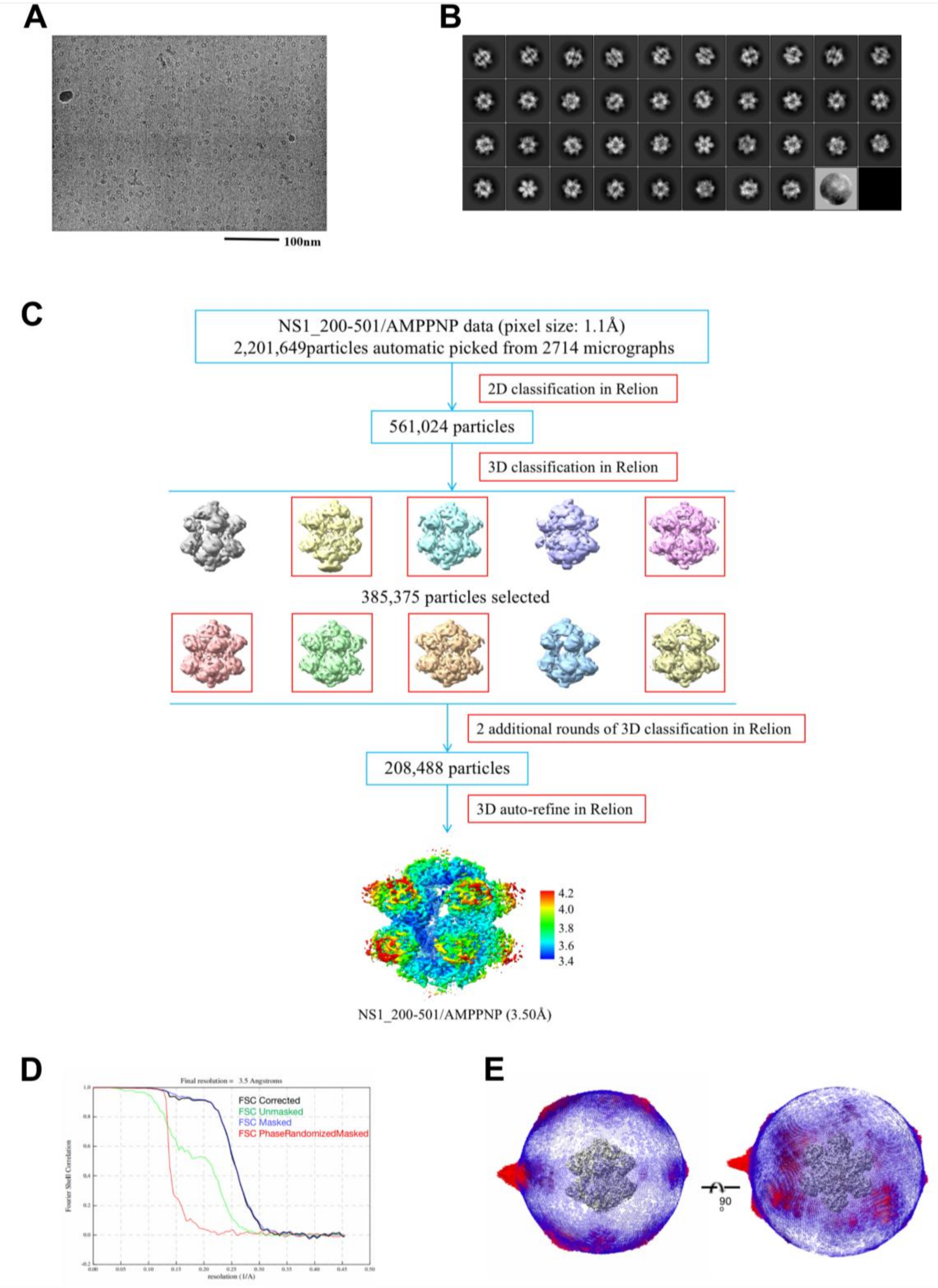

Fig. S18: Cryo-EM data processing of the NS1_200-501/AMPPNP structure. (**A**) A representative cryo-EM image of the NS1_200-501/AMPPNP structure. (**B**) Representative 2D classification images of the NS1_200-501/AMPPNP structure. (**C**)

Flowchart of cryo-EM data processing and final density map colored according to the local resolution. (**D**) Gold-standard Fourier shell correlation (GSFSC) of the final map of the NS1_200-501/AMPPNP structure. (**E**) Particle orientation distribution of the NS1_200-501/AMPPNP structure.

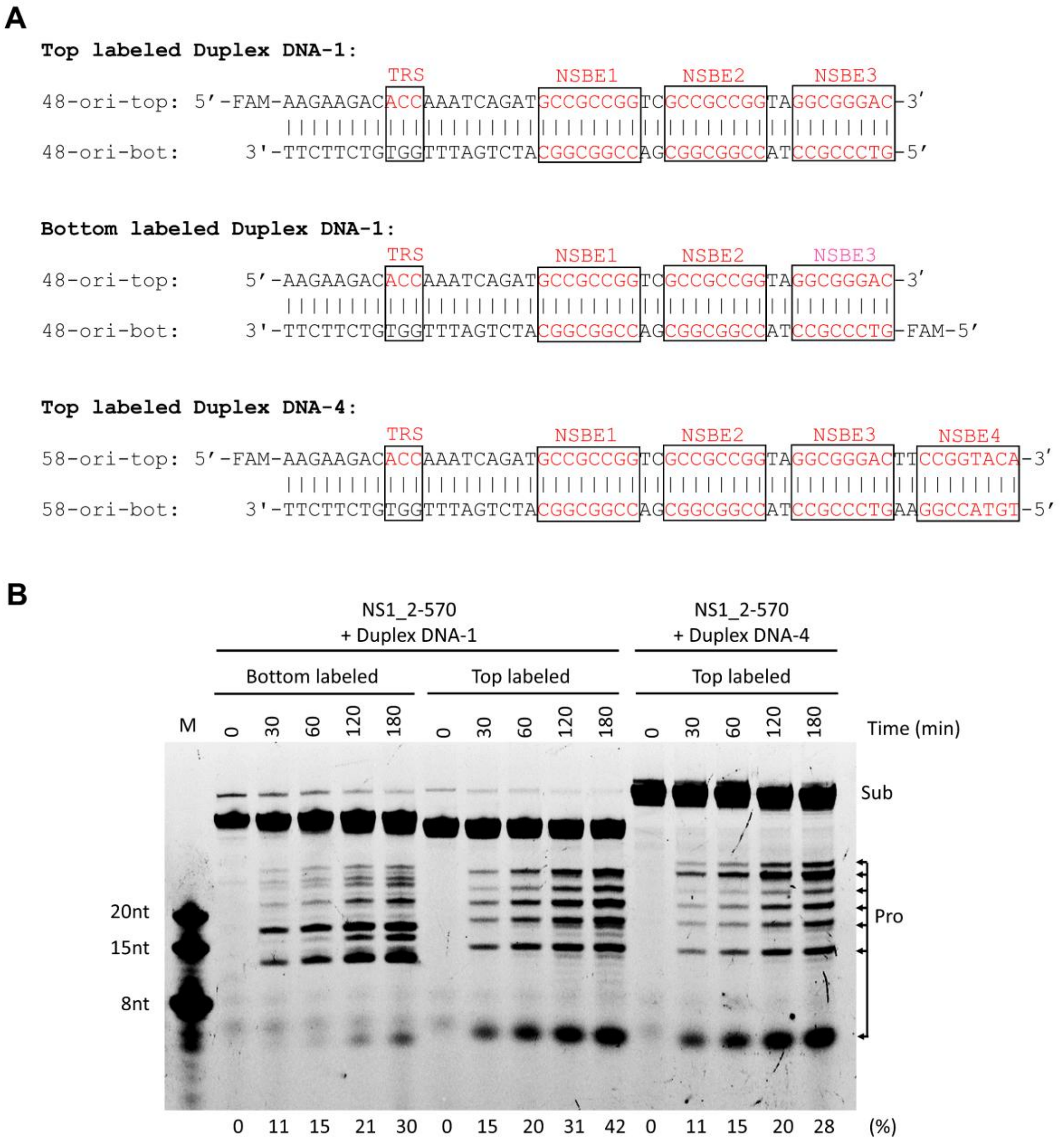

Fig. S19: ***In vitro*** **dsDNA cleavage assays catalyzed by NS1_2-570.** (**A**) Sequences of the DNAs used in the cleavage assays. (**B**) *In vitro* dsDNA cleavage by NS1_2-570. The substrate cleavage percentage (%) is shown at the bottom of the gels. Experiments were repeated independently three times with similar results.

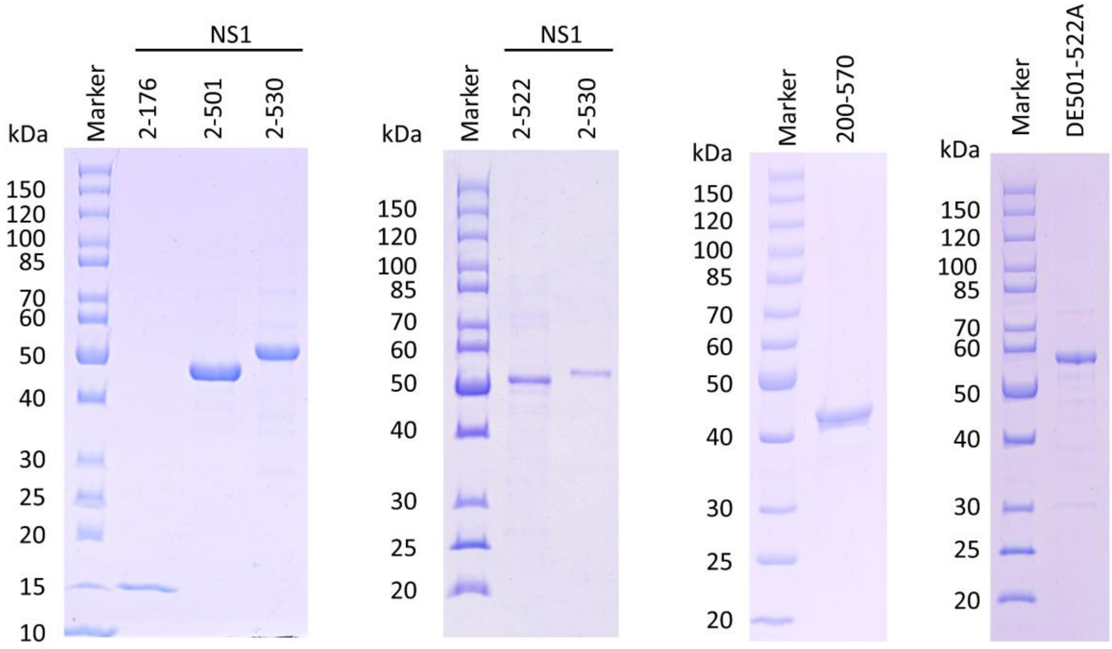

Fig. S20: SDS-page gel analysis of the truncated or mutated NS1 proteins.

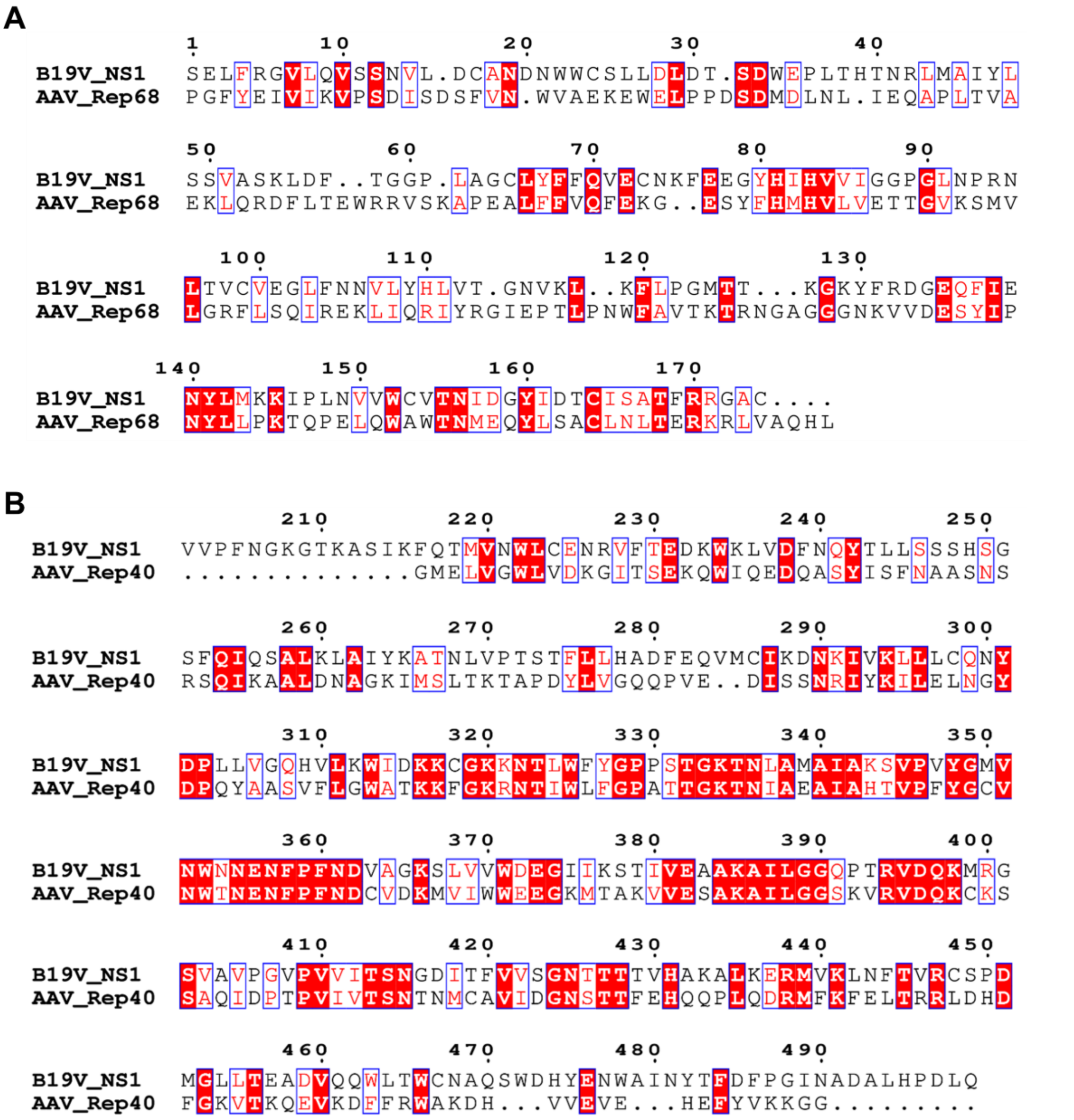

Fig. S21: Sequence alignment of B19V NS1 and AAV Rep proteins. (**A**) Sequence alignment of the Nuc domain. (**B**) Sequence alignment of the OD and SF3 HD domains.

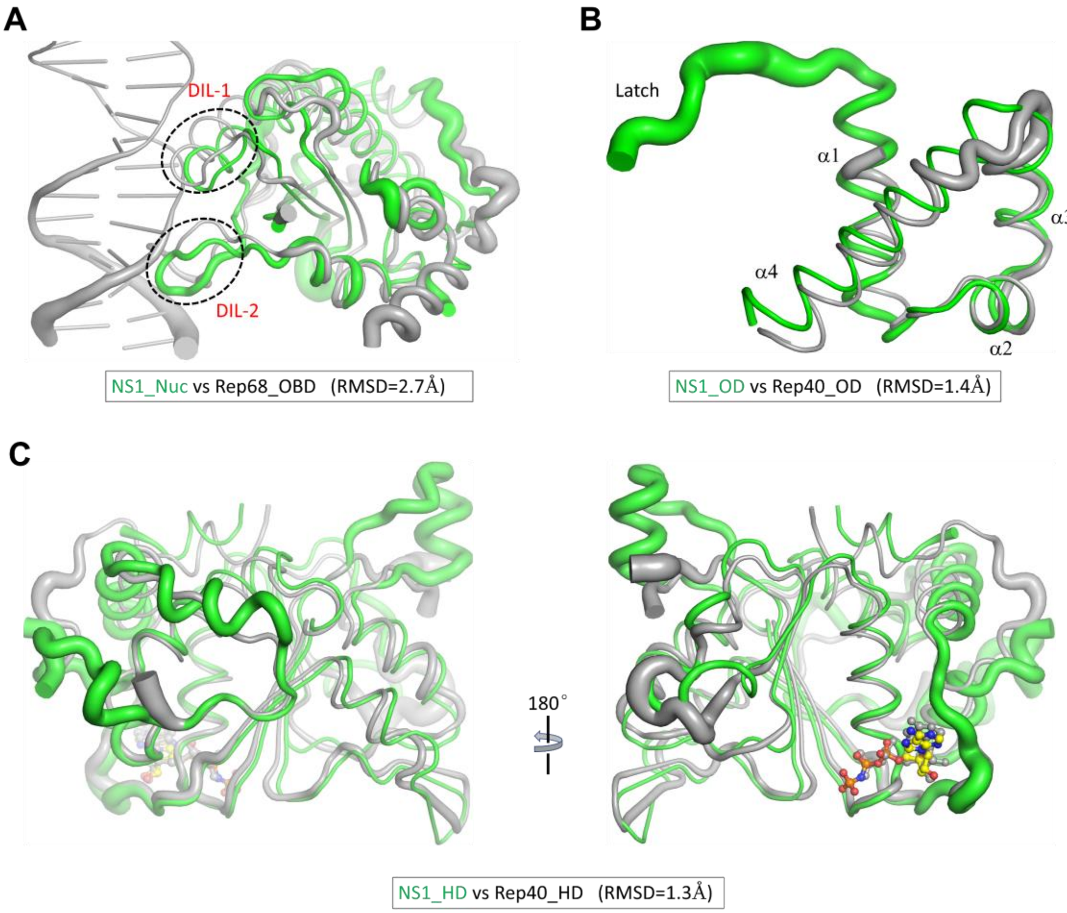

Fig. S22: Structural comparison of B19V NS1 and AAV Rep proteins. (**A**) Superposition of the reported NS1_Nuc structure (PDB_ID: 7Y56) with the DNA-bound ODB domain structure of Rep68 (PDB_ID: 4ZQ9). (**B-C**) Superposition of the OD domain and the SF3 HD domain in B19V NS1 and the ADP-bound AAV Rep40 structure (PDB_ID: 1U0J). Rep proteins and the bound DNA or ADP are colored in gray in all panels. NS1 protein is shown as colored in green, whereas AMPPNP bound by NS1 is shown as sticks in atomic colors (C, yellow; N, blue; O, red; P, orange). Both NS1 and AAV Rep structures are presented in sausage views based on the B factors.

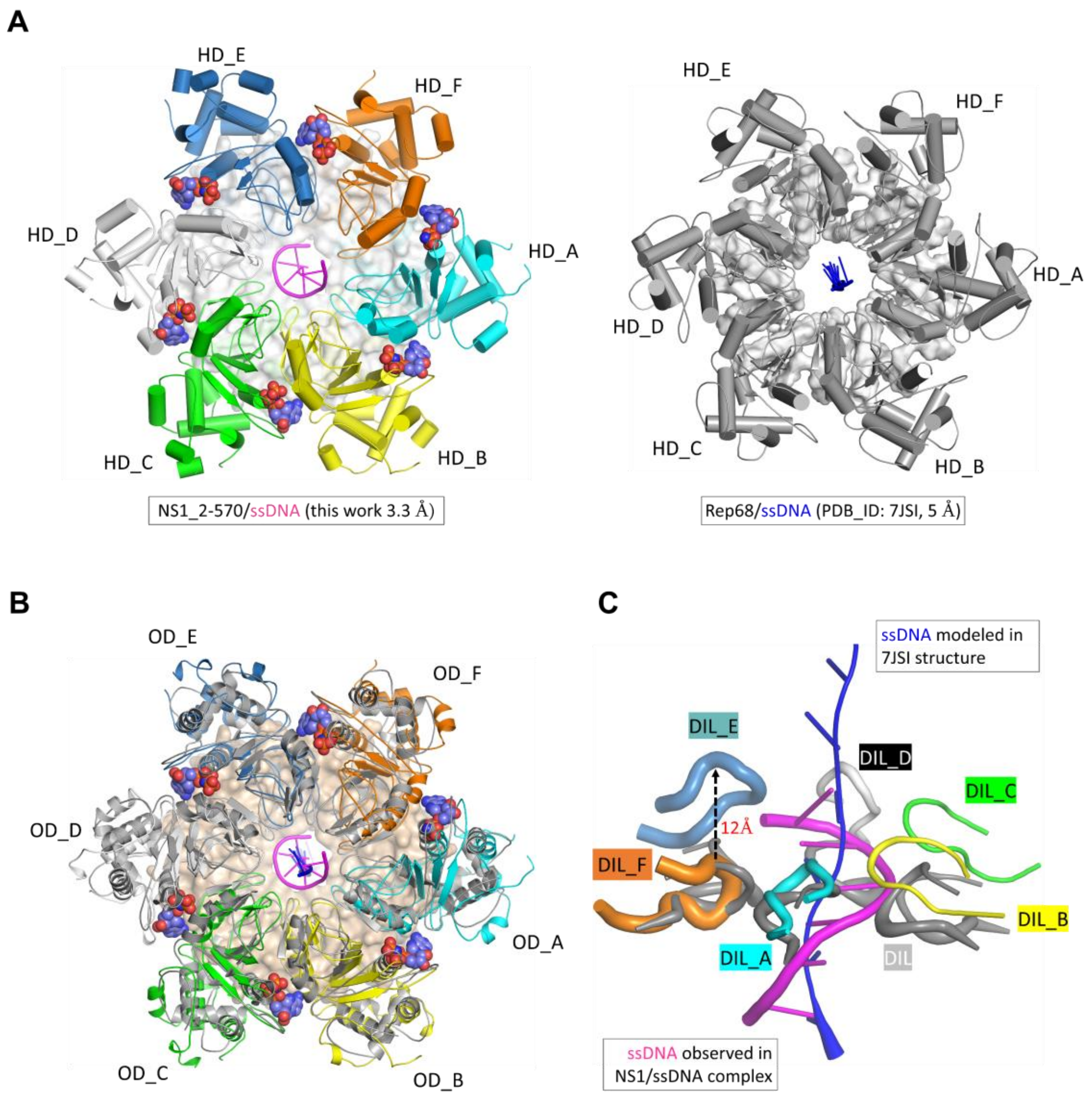

Fig. S23: Structural comparison of ssDNA-bound B19V NS1 and AAV Rep68. (**A**) Overall structures of the ssDNA-bound NS1 structure and the Rep68 protein structure (PDB_ID: 7JSI). (**B**) Superposition of the ssDNA-bound NS1 and Rep68 structure. (**C**) Conformational comparison of the ssDNA and the DNA-interacting loops (DIL) in the NS1 and the Rep68 structures, which are presented in sausage views based on the B factors. For the ssDNA-bound Rep68 structure, the ssDNA and the protein are colored in blue and gray, respectively. For the ssDNA-bound NS1 structure, the ssDNA is colored in magenta, the protomers A to F of NS1 are colored in cyan, yellow, green, gray, light blue, and orange, respectively. The AMPPNP bound by NS1 is shown as spheres in atomic colors (C, light blue; N, blue; O, red; P, orange).

Table S1. Codon optimized cDNA sequence of NS1 (aa 2-570)

The optimized cDNA sequence of NS1 (from 5' to 3') [a,b]
GGATCCGGCGGCGGTGAACTGTTTCGCGGCGTGTTACAGGTGAGTAGCAATGTGCT<br>GGATTGTGCCAATGATAATTGGTGGTGTAGCCTGCTCGACCTGGATACCAGCGATT<br>GGGAACCGTTAACCCATACAAATCGCCTCATGGCCATTTATCTGTCAAGCGTTGCA<br>AGTAAACTGGATTTTACCGGCGGTCCGTTAGCAGGTTGTCTGTATTTTTTTCAGGT<br>GGAATGTAATAAATTTGAAGAAGGCTATCATATTCATGTGGTGATTGGCGGTCCGG<br>GTCTGAATCCGCGCAATCTGACCGTTTGTGTGGAAGGCTTATTTAATAATGTGCTG<br>TATCATTTAGTGACCGGCAATGTTAAACTGAAATTTCTGCCGGGTATGACCACCAA<br>AGGTAAATATTTTCGCGATGGCGAACAGTTTATTGAAAATTATCTGATGAAAAAAA<br>TTCCGCTGAATGTTGTTTGGTGTGTGACCAATATTGATGGCTATATTGATACCTGT<br>ATTAGCGCAACCTTTCGTCGCGGCGCCTGTCATGCCAAAAAACCGCGTATGACCAC<br>CGCCATTAATGATACCTCAAGTGATGCAGGCGAACCGAGCGGCACCGGCGCAGAAG<br>TGGTTCCGTTTAATGGCAAAGGCACCAAAGCCTCTATTAAATTTCAGACTATGGTG<br>AATTGGCTGTGTGAAAATCGCGTGTTTACCGAAGATAAATGGAAACTGGTTGATTT<br>TAATCAGTATACCTTACTGAGTTCATCACATAGCGGTAGCTTTCAGATTCAGAGTG<br>CCCTGAAACTGGCCATTTATAAAGCAACCAATTTAGTTCCGACCTCAACCTTTCTG<br>CTACATGCAGATTTTGAACAGGTTATGTGTATTAAAGATAATAAAATTGTTAAACT<br>GCTGCTGTGTCAGAATTATGATCCGTTACTGGTGGGCCAGCATGTTCTGAAATGGA<br>TTGATAAAAAATGTGGTAAAAAAAATACCCTGTGGTTTTATGGCCCGCCGTCAACC<br>GGCAAAACGAATTTGGCGATGGCCATTGCCAAATCAGTGCCGGTGTATGGCATGGT<br>GAATTGGAATAATGAAAATTTTCCGTTTAATGATGTTGCAGGTAAGTCCTTAGTGG<br>TTTGGGATGAAGGCATTATTAAATCTACCATTGTGGAAGCCGCGAAAGCCATCTTA<br>GGCGGTCAGCCGACCCGCGTTGATCAGAAAATGCGCGGCTCAGTTGCAGTTCCGGG<br>CGTGCCGGTTGTTATTACCTCTAATGGCGATATTACCTTTGTTGTGTCAGGCAATA<br>CCACCACCACCGTTCATGCCAAAGCCCTGAAAGAACGTATGGTTAAACTGAATTTT<br>ACCGTGCGCTGTAGTCCGGATATGGGTCTGCTGACCGAAGCAGATGTTCAGCAGTG<br>GTTAACCTGGTGTAATGCACAGTCTTGGGATCATTATGAAAATTGGGCCATTAATT<br>ATACCTTTGATTTTCCGGGCATTAATGCAGATGCCTTACATCCGGATTTGCAGACC<br>ACCCCGATTGTGACCGATACCTCTATTTCTTCTTCAGGTGGCGAATCAAGCGAAGA<br>ACTGAGCGAATCTAGCTTTTTTAATCTGATTACGCCTGGCGCATGTAATACCGAAA<br>CCCCGCGTAGTAGTACCCCGATTCCGGGCACCAGCTCAGGCGAGAGCTTAGTGGGC<br>TCTCCGGTGAGTAGCGAAGTGGTTGCAGCCTCTTGGGAAGAACTCGAG

[a]: GGATCC and CTCGAG at the 5'-end and 3'-end are Bam HI and Xho I recognition sequence.

[b]: GGCGGCGGT at the 5'-end is designed to encode for three Gly residues.

Table S2. Primers used for NS1 mutant construction

Name	Sequence (from 5' to 3')
2-570_F	GAACTGTTTCGCGGCGTGTTACAGGTGA
2-570_R	TTATTCTTCCCAAGAGGCTGCAACCAC
2-570_T210A_F	TTTAATGGCAAAGGCGCAAAAGCCTCTATTAAATTTCAGACTATGGT
2-570_T210A_R	TTTAATAGAGGCTTTTGCGCCTTTGCCATTAAACGGAACC
2-570_K211A_F	AATGGCAAAGGCACCGCAGCCTCTATTAAATTTCAGACTATGGTGAA
2-570_K211A_R	AAATTTAATAGAGGCTGCGGTGCCTTTGCCATTAAACGGA
2-570_K320A_F	GATAAAAAAATGTGGTGCAAAAAATACCCTGTGGTTT
2-570_K320A_R	CCACAGGGTATTTTTTGCACCACATTTTTTATCAAT
2-570_K334A_F	CCGCCGTCAACCGGCGCAACGAATTTGGCGATGGCCATTGC
2-570_K334A_R	CATCGCCAAATTCGTTGCGCCGGTTGACGGCGGG
2-570_T335A_F	CCGTCAACCGGCAAAGCTAATTTGGCGATGGCCATTGC
2-570_T335A_R	GGCCATCGCCAAATTAGCTTTGCCGGTTGACGGCG
2-570_Q391A_F	GCCATCTTAGGCGGTGCACCGACCCGCGTTGATCAG
2-570_Q391A_R	ATCAACGCGGGTCGGTGCACCGCCTAAGATGGCTTT
2-570_K398A_F	ACCCGCGTTGATCAGGCAATGCGCGGCTCAGTTG
2-570_K398A_R	AACTGAGCCGCGCATTGCCTGATCAACGCGGGTCG
2-570_M399A_F	CGCGTTGATCAGAAAGCTCGCGGCTCAGTTGCAG
2-570_M399A_R	TGCAACTGAGCCGCGAGCTTTCTGATCAACGCGGGTCG
2-570_R438A_F	AAAGCCCTGAAAGAAGCAATGGTTAAACTGAATTTT
2-570_R438A_R	ATTCAGTTTAACCATTGCTTCTTTCAGGGCTTTGGC
DE501-522A_F	ACCGCTACCTCTATTTCTTCTTCAGGTGGCGCATCAAGCGCAGCACTGAGCGCA

DE501-522A_R	AGATGCGCTCAGTGCTGCGCTTGATGCGCCACCTGAAGAAG AAATAGAGGTAGC
2-176_F	GGATCCGGCGGCGGTGAACTGTTTCGCGGCGTGTTACAGGT G
2-176_R	CTCGAGTTAGGCATGACAGGCGCCGCGACGA
200-570_F	GCAGAAGTGGTTCCGTTTAATGGCAAAG
200-570_R	TTATTCTTCCCAAGAGGCTGCAACCAC
2-501_F	GAACTGTTTCGCGGCGTGTTACAGGTGA
2-501_R	TTAGGTGGTCTGCAAATCCGGATGTAAGG
2-522_F	GAACTGTTTCGCGGCGTGTTACAGGTGA
2-522_R	TTATTCGCTCAGTTCTTCGCTTGATTCG
2-530_F	GAACTGTTTCGCGGCGTGTTACAGGTGA
2-530_R	TTACGTAATCAGATTAAAAAAGCTAGATTCGCTCAGT
200-501_F	GCAGAAGTGGTTCCGTTTAATGGCAAA
200-501_R	TTAGGTGGTCTGCAAATCCGGATGTAAG
200-501_K334A_F	CCGCCGTCAACCGGCGCAACGAATTTGGCGATG
200-501_K334A_R	CATCGCCAAATTCGTTGCGCCGGTTGACGGCGG
200-501_3G_F	ATTACCTTTGTTGTGGGTGGAGGCACCACCGTTCATGCCAA A
200-501_3G_R	GGCATGAACGGTGGTGCCTCCACCCACAACAAAGGTAATAT C

Table S3: Cryo-EM data collection, processing, model refinement and validation statistics.

**Structure**	NS1_2-570/ AMPPNP	NS1_2-570/ ssDNA/ AMPPNP	NS1_2-570/ dsDNA/ AMPPNP	NS1_200-501/ AMPPNP
PDB ID	9KBG	9KBH	9KBI	9KBJ
EMDB ID	EMD-62224	EMD-62225	EMD-62226	EMD-62227
**Data collection and Procession**				
Microscope	Krios G4i		Titan Krios	
Detector	Falcon 4i with selectrisX		Gatan K3	
CS (mm)	2.7	2.7	2.7	0.01
Magnification	130K	130K	130K	64K
Pixel size (Å)	0.959	0.959	0.959	1.10
Electron dose (e-/Å^2)	50 (32 frames)	50 (32frames)	50 (32frames)	50 (40frames)
Defocus range (μm)	-1.2 to -2.0	-1.2 to -2.0	-1.2 to -2.0	-1.3 to -1.8
Micrograph Number	5817	5817	7465	2714
**Reconstruction**				
Software	RELION-5.0, CryoSPARC-v4			RELION-3.0
Particles picked	1,814,859	2,078,916	1,814,859	2,201,649
Particles refinement	321,748	25,261	20,491	208,488
Symmetry	C1	C1	C1	C1
Resolution (Å)	2.75	3.3	3.43	3.5
Sharpening B-factor (Å)	105.5	80.2	67.5	122.58
**Refinement**				
Software	Phenix-1.21.1_5286			
Model Composition				
Number of atoms	27690	13770	28248	27366
Protein residues	3491	1737	3482	3430
Nucleotides	0	6	27	0
AMPPNP/Mg^{2+}	12/12	6/4	12/8	11/11
B factors (Å^2)				
Protein/DNA/AMPPNP	53.6/0/66.4	106.8/110.8/111.8	112.9/195.7/139.4	54.6/0/71.0
Bonds RMSD				
Bonds lengths (Å)	0.007	0.006	0.003	0.005
Bonds angels (°)	0.861	0.750	0.578	0.730
**Validation**				
MolProbity score	1.62	1.77	1.32	1.13

Clash score	5.05	5.83	4.57	3.41
Rotamer outliers (%)	1.26	0.82	1.02	0.03
C-beta outliers (%)	0.27	NA	0.03	NA
Ramachandran plot				
Favored (%)	95.96	92.99	97.64	98.75
Allowed (%)	3.28	6.48	1.98	1.25
Outlier (%)	0.76	0.53	0.38	0.00
Model vs. Data				
CC mask/box	0.82/0.70	0.83/0.76	0.82/0.77	0.82/0.79
